# Mechanisms of amphetamine action illuminated through optical monitoring of dopamine synaptic vesicles in *Drosophila* brain

**DOI:** 10.1038/ncomms10652

**Published:** 2016-02-16

**Authors:** Zachary Freyberg, Mark S. Sonders, Jenny I. Aguilar, Takato Hiranita, Caline S. Karam, Jorge Flores, Andrea B. Pizzo, Yuchao Zhang, Zachary J. Farino, Audrey Chen, Ciara A. Martin, Theresa A. Kopajtic, Hao Fei, Gang Hu, Yi-Ying Lin, Eugene V. Mosharov, Brian D. McCabe, Robin Freyberg, Kandatege Wimalasena, Ling-Wei Hsin, Dalibor Sames, David E. Krantz, Jonathan L. Katz, David Sulzer, Jonathan A. Javitch

**Affiliations:** 1 Department of Psychiatry, College of Physicians & Surgeons, Columbia University, New York, New York 10032, USA; 2 Division of Molecular Therapeutics, New York State Psychiatric Institute, New York, New York 10032, USA; 3 Department of Neurology, College of Physicians & Surgeons, Columbia University, New York, New York 10032, USA; 4 Psychobiology Section, Intramural Research Program, Department of Health and Human Services, National Institute on Drug Abuse, National Institutes of Health, Baltimore, Maryland 21224, USA; 5 Department of Biochemistry and Molecular Biophysics, Howard Hughes Medical Institute, College of Physicians & Surgeons, Columbia University, New York, New York 10032, USA; 6 Department of Psychiatry and Biobehavioral Sciences and Semel Institute for Neuroscience and Human Behavior, Hatos Center for Neuropharmacology, David Geffen School of Medicine University of California, Los Angeles, California 90095, USA; 7 UCLA Interdepartmental Program in Molecular Toxicology, University of California, Los Angeles, California 90095, USA; 8 Department of Chemistry, Columbia University, New York, New York 10027, USA; 9 School of Pharmacy, College of Medicine, National Taiwan University, Taipei, Republic of China 10055; 10 Center for Motor Neuron Biology and Disease, College of Physicians & Surgeons, Columbia University, New York, New York 10032, USA; 11 Department of Pathology and Cell Biology, College of Physicians & Surgeons, Columbia University, New York, New York 10032, USA; 12 Department of Neuroscience, College of Physicians & Surgeons, Columbia University, New York, New York 10032, USA; 13 Department of Psychology, Stern College for Women, Yeshiva University, New York, New York 10016, USA; 14 Department of Chemistry, Wichita State University, Wichita, Kansas 67260, USA; 15 Department of Pharmacology, College of Physicians & Surgeons, Columbia University, New York, New York 10032, USA

## Abstract

Amphetamines elevate extracellular dopamine, but the underlying mechanisms remain uncertain. Here we show in rodents that acute pharmacological inhibition of the vesicular monoamine transporter (VMAT) blocks amphetamine-induced locomotion and self-administration without impacting cocaine-induced behaviours. To study VMAT's role in mediating amphetamine action in dopamine neurons, we have used novel genetic, pharmacological and optical approaches in *Drosophila melanogaster*. In an *ex vivo* whole-brain preparation, fluorescent reporters of vesicular cargo and of vesicular pH reveal that amphetamine redistributes vesicle contents and diminishes the vesicle pH-gradient responsible for dopamine uptake and retention. This amphetamine-induced deacidification requires VMAT function and results from net H^+^ antiport by VMAT out of the vesicle lumen coupled to inward amphetamine transport. Amphetamine-induced vesicle deacidification also requires functional dopamine transporter (DAT) at the plasma membrane. Thus, we find that at pharmacologically relevant concentrations, amphetamines must be actively transported by DAT and VMAT in tandem to produce psychostimulant effects.

## Abstract (web summary)

Amphetamines are known to enhance extracellular dopamine levels, but the underlying mechanisms are unclear. Utilising a new pH biosensor for synaptic vesicles, the authors show that amphetamines diminish vesicle pH gradients, disrupting dopamine packaging and leading to increased neurotransmitter release.

## Graphical abstract

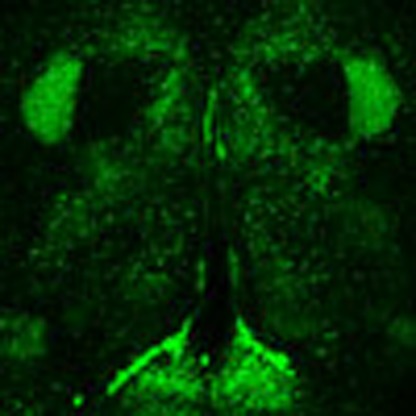

Prescribed and illicit amphetamines, including amphetamine and methamphetamine derivatives, are some of the most widely used and abused drugs: Total prescriptions number over 15 million yearly in the US, with ∼56 million users globally^1^. Amphetamines' psychostimulant effects are generally thought to result from increased extracellular dopamine mediated by efflux of cytoplasmic dopamine through the dopamine transporter (DAT)^2^. How amphetamines mobilize dopamine from vesicles to the cytoplasm for subsequent efflux is less clear. Dopamine is synthesized in the cytosol and concentrated into synaptic vesicles ∼10^5^-fold (∼0.1 M intraluminal dopamine) relative to cytoplasmic dopamine (∼1 μM) by the vesicular monoamine transporter (VMAT)^3^,^4^,^5^. Whether amphetamines also act directly on VMAT to redistribute dopamine from vesicles into the cytoplasm has been debated, and numerous mechanisms have been proposed^6^.

Amphetamines interact with VMAT *in vitro*, leading some investigators to conclude that they act as non-substrate inhibitors that elevate cytoplasmic dopamine by simply blocking its accumulation into vesicles and thus making more available for efflux^7^. Others have inferred that amphetamines are substrates of VMAT that drive carrier-mediated exchange of vesicular monoamines into the cytoplasm^8^,^9^. A third proposed mechanism is that amphetamines deplete synaptic vesicle dopamine stores through mechanisms akin to lipophilic weak bases and protonophores^2^,^10^, which degrade the vesicular pH gradient (ΔpH). This ΔpH, generated by the vacuolar H^+^-ATPase (V-ATPase), not only drives loading of monoamines into the vesicle lumen by VMAT, but also promotes intravesicular retention of monoamines through their protonation^11^. Amphetamines are weak bases (pK_a_ 8.8–9.9) and have been proposed to decrease the vesicular ΔpH by buffering luminal free protons, thereby depleting vesicles of stored dopamine and indirectly blocking substrate import. This is the mechanism of action for weak bases such as chloroquine, which is lipophilic enough to diffuse across lipid bilayers (Log*P*=4.72)^11^,^12^,^13^,^14^. Although amphetamines are less lipophilic (for example, Log*P* of amphetamine=1.41)^15^, they have been hypothesized both to enter vesicles by diffusion as well as by VMAT-mediated transport^2^. Evidence for these diverse mechanisms has come in large part from *in vitro* studies of isolated vesicles, cells and brain slices, but the actual relevance of these proposed mechanisms to amphetamines' *in vivo* actions has not been ascertained.

Earlier work by Dwoskin and colleagues showed that tetrabenazine and lobeline analogues, which are inhibitors of the neuronal VMAT isoform, VMAT2, blocked methamphetamine's behavioural action in rodents^16^,^17^. While these results suggested that VMAT is instrumental in mediating methamphetamine's effects, several of these compounds have modest selectivity for VMAT2 and also inhibit DAT. To address this, we developed a new VMAT blocker, (+)-CYY477, with improved selectivity and show that acute VMAT inhibition in rodents blocks locomotor and self-administration behaviours stimulated by amphetamines without affecting those induced by cocaine. This selective antagonism indicates that VMAT function is required for the acute actions of amphetamines to release dopamine from intraluminal stores.

To elucidate how amphetamines act on synaptic vesicles to release dopamine into the cytoplasm, we developed an experimental system in *Drosophila melanogaster* using a functionally viable *ex vivo* brain preparation^16^. Actions of amphetamines were studied in this whole-brain preparation using small synthetic and genetically-encoded fluorescent reporters visualized by multiphoton microscopy. We used the second-generation fluorescent false neurotransmitter FFN206 (ref. 17) in combination with genetic manipulations to monitor in real time the dynamics of dopaminergic vesicle cargo in whole fly brains. To examine the effects of amphetamines on vesicle pH, we expressed the synaptic vesicle pH biosensor, dVMAT-pHluorin^18^, in dopaminergic neurons, which allowed us to monitor real-time changes in synaptic vesicle pH in whole living brain.

Our findings in flies demonstrate that at pharmacologically relevant concentrations, amphetamines, as well as other VMAT substrates, must be transported by VMAT to diminish vesicular ΔpH and redistribute vesicular contents. Our data suggest that VMAT-mediated substrate-coupled H^+^ antiport provides the critical mechanism for amphetamine-induced vesicular deacidification.

## Results

### VMAT mediates acute amphetamine-induced rodent behaviours

**Figure 1:**
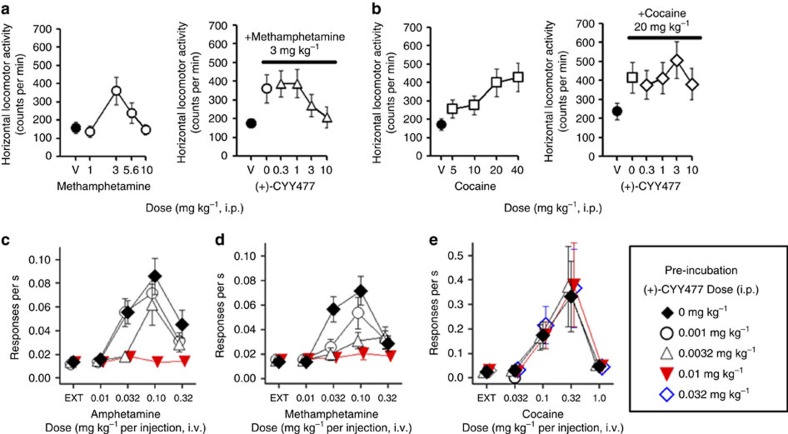
Acute (+)-CYY477 pretreatment selectively antagonizes behavioural effects of amphetamine and methamphetamine in rodents. (**a**) Methamphetamine stimulation of horizontal locomotion in mice. Left panel: methamphetamine increased activity in a dose-related manner with maximal elevation at a 3 mg kg^−1^ i.p. dose (open points). ‘V' (vehicle) indicates locomotor activity rate after two saline vehicle injections (closed point). Right panel: locomotion stimulated by 3 mg kg^−1^ methamphetamine (solid bar) was antagonized by acute preinjection of (+)-CYY477 in a dose-dependent manner (open triangles) (**b**) Cocaine stimulation of horizontal locomotion in mice. Left panel: dose-related increases in activity (open squares, ‘V' two saline injections). Right panel: Locomotion stimulated by 20 mg kg^−1^ cocaine (solid bar) was unaffected by acute preinjection of (+)-CYY477 up to 10 mg/kg doses (open diamonds). (**a**,**b**) all drug doses were administered i.p. Mean and s.e.m. from six mice per group. (**c**–**e**) Selective antagonism by (+)-CYY477 of self-administration of psychostimulants in rats. Acute (+)-CYY477 pretreatment dose-dependently attenuated self-administration of both amphetamine (**c**) (*P*<0.001) and methamphetamine (**d**) (*P*<0.001). For self-administration rates (0.10 mg kg^−1^ per injection of either amphetamine): (+)-CYY477 0.001 mg/kg (*t*⩾2.33; *P*≤0.14), 0.0032, mg kg^−1^ (*t*⩾3.87; *P*≤0.002), 0.01 mg kg^−1^ (*t*⩾9.03; *P*<0.001). Two-way repeated measure ANOVAs also indicate significant effects on amphetamine doses (F(4,60)=21.8, *P*<0.001) and methamphetamine doses (F(4,60)=23.6, *P*<0.001). (**e**) Cocaine self-administration was not diminished by acute (+)-CYY477 pretreatment at any dose tested. (**c**–**e**) Ordinates: responses per s; abscissae: psychostimulant dose ( mg kg^−1^ per injection, i.v.). Each point represents the mean±s.e.m. of response rates on the active lever in six rats per group; EXT: extinction (no injection). ‘0 mg/kg' of (+)-CYY477 indicates vehicle (saline-only) pretreatment. i.p., intraperitonially; i.v., intravenous.

To validate VMAT2's role in acute behavioural actions of amphetamines in rodents, we developed (+)-CYY477, a novel derivative of dihydrotetrabenazine that retains high VMAT2 affinity but has better selectivity (Supplementary Note 1). (+)-CYY477 potently inhibited dopamine uptake by rat VMAT2 expressed in cultured cells (half-maximal inhibitory concentration (IC_50_)=26 nM; Supplementary Fig. 1a). (+)-CYY477 also exhibited good selectivity for vesicular over plasma membrane monoamine transporters. It inhibited binding to mammalian VMAT2 (*K*_i_=7.18±1.14 nM), but was inactive at DAT, the serotonin transporter and the norepinephrine transporter (see Supplementary Methods, Supplementary Table 1). Thus, given (+)-CYY477's potency and selectivity for VMAT2, we used it to examine effects of acute pharmacological inhibition of VMAT2 on psychostimulant actions in both mice and rats (see Supplementary Methods, Fig. 1, Supplementary Fig. 2). In these protocols, (+)-CYY477 was administered ≤5 min preceding psychostimulant treatments, allowing us to avoid potential confounds associated with gene knockdown or chronic pharmacological depletion of monoamine stores. Acute (+)-CYY477 pretreatment (0.3–10 mg kg^−1^) significantly diminished methamphetamine (3 mg kg^−1^)-induced hyperlocomotion in mice (Fig. 1a). In contrast, (+)-CYY477 failed to attenuate cocaine (20 mg kg^−1^)-induced hyperlocomotion (Fig. 1b). Preservation of the locomotor response to cocaine indicates that vesicular dopamine stores were functionally intact using these (+)-CYY477 doses. Notably, the acute (+)-CYY477 doses used in this mouse hyperlocomotion assay were substantially lower than those that produced catalepsy (Supplementary Fig. 1b, Supplementary Discussion). Furthermore, in psychostimulant self-administration assays performed in rats, acute (+)-CYY477 pretreatment inhibited self-administration of both methamphetamine and amphetamine in a dose-dependent manner (Fig. 1c,d), whereas cocaine self-administration was again unaffected (Fig. 1e; Supplementary Discussion). Both cocaine and amphetamines are expected to produce locomotor and self-administration behaviours through the common step of elevating extracellular dopamine levels^19^. However, that (+)-CYY477 selectively antagonized amphetamine and methamphetamine-induced behaviours but not those of cocaine, clearly illustrates that VMAT2 plays a critical role in the acute actions of amphetamines but not those of cocaine.

### dVMAT is essential for amphetamine behaviour in *Drosophila*

**Figure 2:**
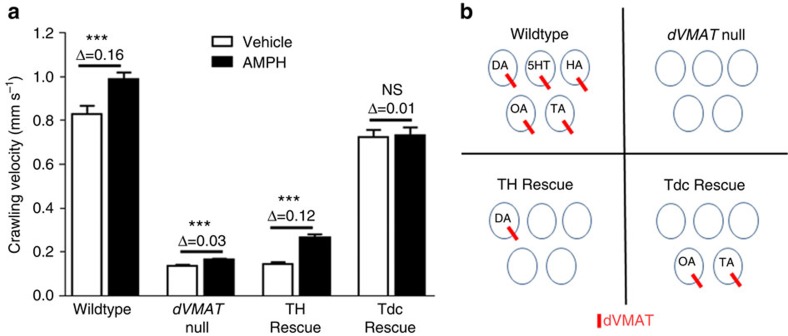
dVMAT expression in presynaptic dopamine neurons mediates amphetamine-induced *Drosophila* larval hyperlocomotion. (**a**) Wildtype (WT) wCS10 larvae fed amphetamine (60 mM) exhibited hyperlocomotion compared with vehicle (yeast alone; Δ mean velocity=0.16 mm s^−1^, *P*=0.002, vehicle: *N*=20; amphetamine: *N*=21). *dVMAT* null larvae exhibited significantly diminished baseline crawling velocity relative to the WT controls (5.3-fold difference, *P*<0.001) with a much smaller increase in locomotion velocity to amphetamine (Δ=0.03 mm s^−1^, *t*(95)=4.36, *P*<0.001; vehicle: *N*=51; amphetamine: *N*=46). *dVMAT* null larvae with selective rescue of dVMAT expression in presynaptic dopamine neurons (TH Rescue) exhibited blunted basal locomotion, similar to the *dVMAT* null, but displayed a robust amphetamine response (Δ=0.12 mm s^−1^, *P*<0.001; vehicle: *N*=44; amphetamine: *N*=49). Selective dVMAT rescue in OA/TA neurons (Tdc Rescue) restored basal locomotion to WT levels but not amphetamine-stimulated hyperlocomotion (Δ=0.01 mm s^−1^, *P*>0.05; *N*=44). See Supplementary Fig. 3 showing that expression drivers alone had no effect. Every condition represents the mean±s.e.m. with all experiments conducted on ⩾2 separate occasions. (**b**) Schematic illustration of monoamine neurotransmitter-containing vesicles in flies of different genetic backgrounds. dVMAT expression (indicated in red) confers the ability to accumulate dopamine, OA, TA, serotonin (5-HT) and histamine (HA) in synaptic vesicles but is absent in the *dVMAT* null genetic background. TH Rescue or Tdc Rescue selectively restores dVMAT function in dopamine or OA/TA neurons, respectively.

Building on these findings in rodents, we established a novel experimental system in *D. melanogaster* to dissect mechanisms of monoaminergic neurotransmission *in vivo.* The genetic tractability of *Drosophila* permits targeted manipulation of gene expression to determine contributions of individual genes to amphetamine's actions and the neuronal pathways in which they function. Using a behavioural assay of amphetamine-induced hyperlocomotion^20^,^21^, we first examined whether the sole *Drosophila* VMAT isoform, dVMAT, is required for amphetamine to produce its behavioural effects in larvae. Vehicle-fed *dVMAT* null mutants had significantly diminished baseline crawling velocity relative to wildtype (WT) controls (approximately sixfold decrease, *P*<0.001; Fig. 2a). The amphetamine-induced increase in crawling velocity was approximately fivefold greater in WT than in *dVMA*T null mutants, demonstrating that VMAT is essential for amphetamine-induced hyperlocomotion.

To identify the presynaptic neuronal populations that mediate dVMAT's contribution to amphetamine-induced locomotion, we restricted dVMAT expression to different monoaminergic populations. We used the tyrosine hydroxylase (TH)-GAL4 expression driver^22^ in the *dVMAT* null^23^ background to restore expression of dVMAT selectively in dopamine neurons (‘TH Rescue'; see Fig. 2b schematic). These larvae had a baseline crawling velocity (0.14 mm s^−1^) similar to *dVMAT* null larvae (0.13 mm s^−1^; *P*>0.05 difference) (Fig. 2a). Nonetheless, feeding amphetamine to TH Rescue larvae greatly increased their crawling velocity (Δ=0.12 mm s^−1^; *P*<0.001), producing an increase similar to WT larvae (Δ=0.16 mm s^−1^). dVMAT was also selectively expressed in octopamine (OA) and tyramine (TA) neuron populations using the tyrosine decarboxylase 2 (Tdc2)-GAL4 expression driver^24^ (Tdc Rescue, Fig. 2b) in the *dVMAT* null background. In contrast to TH Rescue animals, Tdc Rescue larvae exhibited no significant amphetamine-stimulated hyperlocomotion (Δ=0.01 mm s^−1^, *P*>0.05) even though their rates of basal locomotion (0.73 mm s^−1^) were comparable to WT (0.83 mm s^−1^; *P*>0.05 difference). Thus, dVMAT expression specifically in dopamine neurons plays a critical role in mediating amphetamine-stimulated behaviour in flies, consistent with voluminous data that dopamine is the primary mediator of amphetamine's psychomotor stimulatory action in mammals^2^,^25^.

### FFN206 labels monoaminergic nerve terminals in whole brain

**Figure 3:**
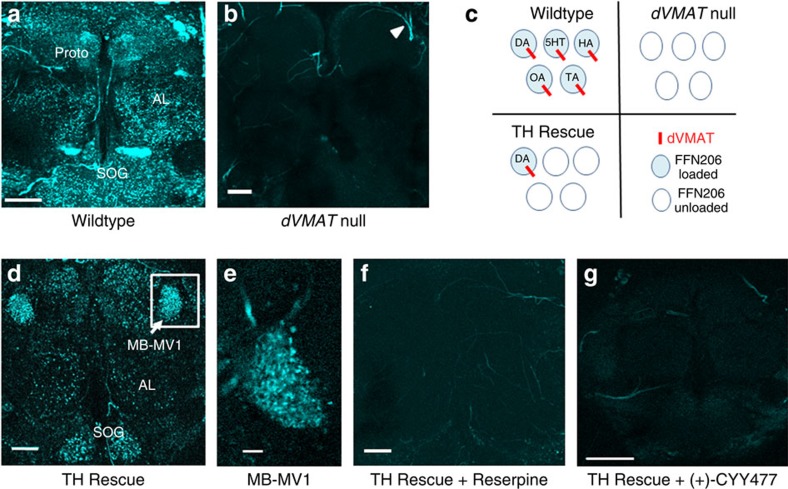
FFN206 accumulation is dependent on dVMAT and selectively labels presynaptic DA nerve terminals in adult fly TH Rescue brain. (**a**) Multiphoton microscopy of FFN206-loaded adult WT brain revealed extensive presynaptic monoaminergic nerve terminal labelling in the central brain neuropil, accumulating in puncta <1 μm diameter. Labelled regions include the suboesophageal ganglion (SOG), antennal lobe (AL) and the protocerebrum (Proto). (**b**) Absence of dVMAT expression in brains of *dVMAT* null mutants abolished virtually all neuropil FFN206 labelling. Residual signal was primarily autofluorescence from tracheal structures (triangle)^74^ as well as some variable staining outside the neuropil. (**c**) Schematic illustration of the expected monoamine content of FFN206-loaded vesicles (blue fill) in WT, *dVMAT* null and TH Rescue brains. (**d**) FFN206 selectively labelled presynaptic dopamine nerve terminals in adult TH Rescue brain with particular enrichment in MB-MV1 neurons (arrow and white box). (**e**) Single-plane image of MB-MV1 as labelled by FFN206 in a TH Rescue brain. (**f**) Reserpine pretreatment of an adult TH Rescue brain prevented FFN206 labelling, similar to *dVMAT* null brain (**b**). (**g**) FFN206 labelling of adult TH Rescue brain was similarly blocked by pretreatment with (+)-CYY477 (1 μM, 20 min, 25 °C) (compare with **f**). String-like structures in **e**–**g** are autofluorescent trachea. Comparable results were obtained from *N*⩾3 independent experiments. Images are from projected Z series of coronal sections acquired with similar settings and of comparable depth; all scale bars are 25 μm, except for **e** (Scale bar, 5 μm). *λ*_ex_=820 nm, *λ*_em_=460/50 nm FWHM.

To analyse the mechanisms of monoamine loading and release from synaptic vesicles, we used an *ex vivo* whole fly brain preparation to optically monitor monoaminergic vesicle content by multiphoton microscopy. We utilized FFN206, which was designed as a fluorescent VMAT2 substrate and has an apparent transport affinity (*K*_m_=1.16±0.10 μM)^17^ similar to that of dopamine (*K*_m_=0.92±0.05 μM) (ref. 8). In WT brains continuously bathed in FFN206, we observed a dense pattern of ∼1 μm fluorescent puncta that varied through the neuropil, consistent with the accumulation of the probe into monoaminergic nerve terminals (Fig. 3a). Labelling was observed in the antennal lobes, suboesophageal ganglion and the protocerebrum (Fig. 3a). Steady-state labelling with 300 nM FFN206 yielded a high signal to background ratio of 60.2±4.7. Neuropil labelling was dVMAT-specific as it was virtually absent from *dVMAT* null mutant flies (Fig. 3b), establishing FFN206 as an excellent tool for labelling monoaminergic vesicles in the fly.

### Selective labelling of dopamine terminals with FFN206

Since FFN206 labels multiple monoaminergic populations that express dVMAT in WT brain, we used the TH Rescue genetic background to drive dVMAT expression selectively in dopamine neurons (Fig. 3c schematic). We observed strong FFN206 labelling of TH Rescue brains in a dense field of dopamine nerve terminals belonging to the MB-MV1 neurons that project to the mushroom bodies and have recently been implicated in associative learning^26^,^27^ (Fig. 3d,e). FFN206 also labelled presynaptic dopamine terminals in the suboesophageal ganglion, antennal lobes, mushroom bodies (Fig. 3d) and the central complex (data not shown). Pretreatment with the VMAT inhibitors reserpine (1 μM, Fig. 3f) or (+)-CYY477 (1 μM, Fig. 3g), completely blocked neuropil labelling by subsequent application of FFN206 in TH Rescue brains. These data are consistent with the necessary role of VMAT for FFN206 labelling, also shown in the genetic *dVMAT* null mutants (Fig. 3b). Therefore, by using FFN206 to label TH Rescue brains, we can specifically image dopamine terminals to examine the neurochemical mechanisms that regulate the content of dopamine vesicles in response to both exocytic and non-exocytic releasing stimuli.

**Figure 4:**
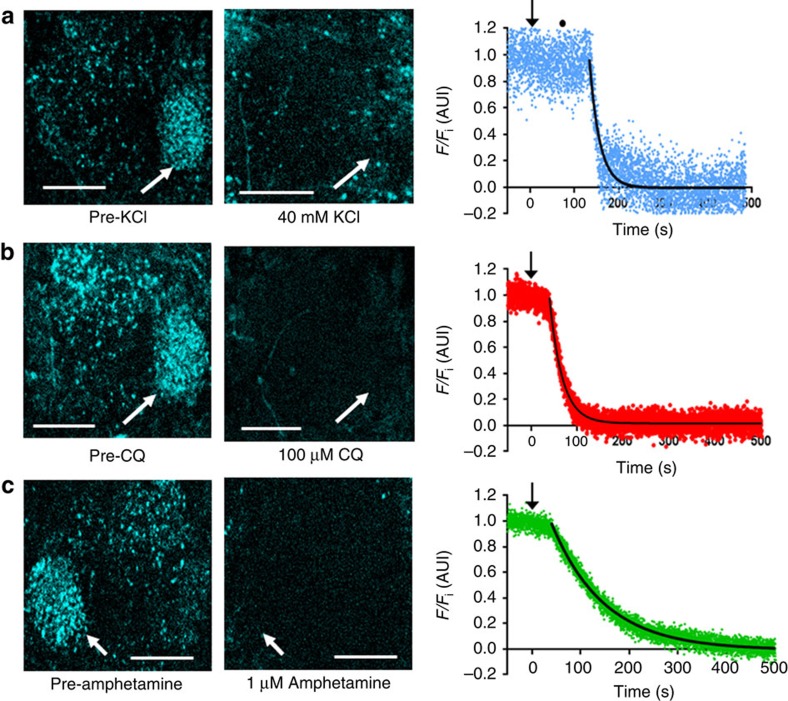
Destaining of FFN206 from dopamine terminals in TH Rescue brains. Fly brains were loaded to steady-state with FFN206 and treated with KCl, chloroquine (CQ), or amphetamine in the continued presence of FFN206. Projected image stacks of the protocerebral neuropil before (left) and after (center) treatments and kinetics of fluorescence decay in MB-MV1 region (right) from representative experiments. (**a**) KCl (40 mM) depolarization caused rapid FFN206 destaining in presynaptic dopamine nerve terminals. Fluorescence intensity is described by an initial plateau followed by a monoexponential decay (black line; for *n*=3 experiments, *t*_1/2_=6.42±0.60 s, *R*^2^=0.85) (**b**) Disrupting the synaptic vesicle pH gradient with chloroquine (100 μM) destained FFN206, and was best fit to an initial plateau followed by a monoexponential decay (black trace; for *N*=4 experiments, *t*_1/2_=26.57±3.73 s, *R*^2^=0.97). (**c**) Amphetamine (1 μM) also caused FFN206 destaining, and was best fit to an initial plateau followed by a monoexponential decay (black trace; for *N*=5 experiments, *t*_1/2_=66.60±17.08 s, *R*^2^=0.99). (**a**–**c**) *λ*_ex_=820 nm, *λ*_em_=460/50 nm FWHM. Fluorescence was normalized to levels attained following FFN206 loading to steady-state with 300 nM as measured in arbitrary units of fluorescence intensity (AUI). Black arrows indicate initiation of drug addition (also containing 300 nM FFN206) and the black circle in **a** represents the point at which 50% of drug reaches the imaging chamber. Scale bar, 25 μm.

To study the kinetics of stimulated release in presynaptic dopamine nerve terminals, we first measured exocytic vesicle release from FFN206-labelled dopamine terminals in TH Rescue brains. KCl-induced depolarization led to pronounced FFN206 destaining from a stable baseline, despite continued FFN206 application during KCl treatment (Fig. 4a). Analysis of destaining kinetics revealed a rapid monoexponential decay of FFN206 signal in the MB-MV1 region (*t*_1/2_=6.42±0.60 s, *R*^2^=0.85±0.04, Fig. 4a). The initial lag before destaining was mainly attributable to the time needed for the KCl to reach the imaging chamber and for solution exchange (Supplementary Fig. 4). The destaining rate is comparable to the rate of exocytic vesicular neurotransmitter release evoked by electrical stimuli at the larval neuromuscular junction (*t*_1/2_=4.49±0.61 s, Supplementary Fig. 5), as well as that described previously using other optical reporters of exocytic neurotransmitter release both in fly neuromuscular junction and cultured vertebrate neurons^28^,^29^. The sparse punctate staining resistant to KCl-depolarization may be due to the heterogeneous release properties of monoaminergic nerve terminals, as reported recently in mouse brain slices^30^.

The lipophilic weak base chloroquine (100 μM) also caused monoexponential destaining of FFN206 (*t*_1/2_=26.57±3.73 s, *R*^2^=0.97±0.01) (Fig. 4b). Treatment with 1 μM amphetamine likewise diminished FFN206 signal to levels comparable to surrounding neuropil background (Fig. 4c) (*t*_1/2_=66.60±17.08 s, *R*^2^=0.99±0.004). Notably, amphetamine-induced FFN206 destaining was not blocked by inhibiting voltage-gated Na^+^ channels (tetrodotoxin, 500 nM) and nicotinic cholinergic receptors (tubocurarine, 10 μM; data not shown), suggesting that the FFN redistribution was due to local action at the terminals and not to circuit-wide excitation. Thus, FFN206 has permitted us to monitor for the first time the dynamics of neurotransmitter content within intact dopaminergic vesicles in an *ex vivo* whole-brain preparation. We hypothesized that KCl, chloroquine and amphetamine operate through distinct mechanisms, and explored these mechanisms with the recently developed *in vivo* pH sensor dVMAT-pHluorin^18^, as described below.

### Characterization of dVMAT-pHluorin, a vesicular pH biosensor

We characterized a genetically encoded fluorescent reporter of intraluminal pH to examine changes in monoamine vesicle pH. This biosensor (termed dVMAT-pHluorin) was created by inserting pHluorin, a superecliptic, pH-sensitive GFP^31^, into the dVMAT polypeptide's first luminal loop^18^. dVMAT-pHluorin was trafficked appropriately to synaptic vesicles as indicated by co-localization with synaptotagmin 1 (syt1), an established vesicle marker (Supplementary Fig. 5a–c). When driven by the TH promoter, dVMAT-pHluorin's expression pattern in presynaptic dopamine nerve terminals (Supplementary Fig. 5g) appeared identical to FFN206's punctate staining pattern in TH Rescue brain (compare with Fig. 3d). dVMAT-pHluorin's fluorescence intensity was dependent on pH, with a high signal to noise ratio (162.6±27.2) and broad dynamic range (Supplementary Fig. 6). Both electrical stimulation (Supplementary Fig. 5d–f) and KCl-induced depolarization significantly enhanced dVMAT-pHluorin's fluorescence intensity by shifting the pH sensor moiety from the acidic vesicle environment to the neutral extracellular space on vesicle exocytosis (Supplementary Fig. 6a). Under basal conditions, the percentage of dVMAT-pHluorin on the cell surface in our *ex vivo* whole fly brain preparation (14.5%±2.5%) is consistent with that described previously for vesicular pH biosensors heterologously expressed in cultured cells^31^,^32^,^33^. dVMAT-pHluorin's pK_a_ (7.5±0.2; Supplementary Fig. 6b) was similar to those of synaptopHluorin (7.1–7.3) and superecliptic pHluorin (7.2)^31^,^34^. Furthermore, dVMAT-pHluorin is a functional transporter as expression of the *UAS-dVMAT-pHluorin* transgene using the Tdc2-GAL4 expression driver in *dVMAT* null background flies rescued basal locomotion to 71% of WT levels (*P*<0.001) (Supplementary Fig. 3b).

### Amphetamine needs DAT and VMAT to alkalize dopamine vesicles

**Figure 5:**
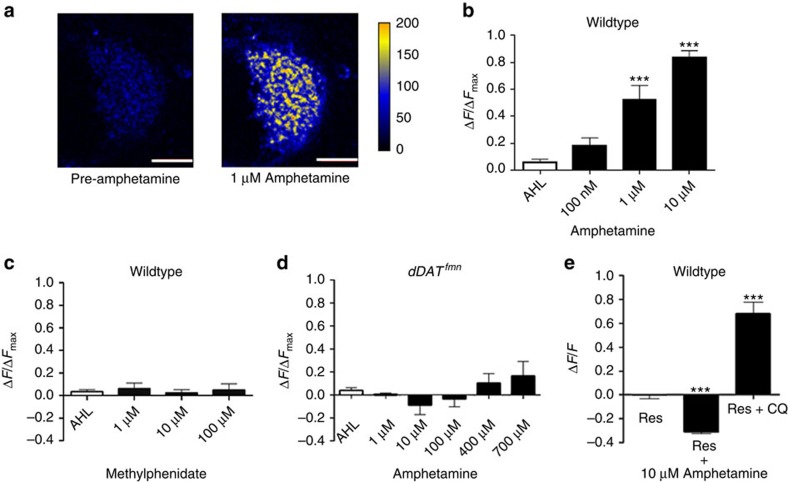
dVMAT-pHluorin, a reporter of intravesicular pH, indicates that functional DAT and VMAT are both required for amphetamine to alkalize vesicles in DA nerve terminals. dVMAT-pHluorin was expressed in dopamine neurons in a WT background (**a**–**c**,**e**) or in the *dDAT* null background (**d**). (**a**) Amphetamine (1 μM) treatment produced significant alkalization of synaptic vesicles as indicated by dVMAT-pHluorin brightening in the MB-MV1 region. Images are from projected Z-series (Scale bar, 10 μm; false colour, arbitrary fluorescence intensity units). (**b**) Increasing doses of amphetamine caused concentration-dependent dVMAT-pHluorin brightening compared with vehicle (AHL) (F(3, 16)=29.96, *P*<0.001). (**c**) Escalating doses of methylphenidate (1–100 μM), a DAT blocker, did not alter vesicular pH (*P*>0.05). (**d**) The *dDAT fumin* (*dDAT*^*fmn*^)null genetic background prevented escalating doses of amphetamine up to 700 μM from causing significant dVMAT-pHluorin brightening. Comparison with the WT genetic background (**b**) shows the vast shift in amphetamine potency due to the absence of functional dDAT in *dDAT*^*fmn*^ mutant brains. (**e**) dVMAT blockade by the continuous presence of reserpine (1 μM) prevented dVMAT-pHluorin brightening by amphetamine (10 μM) but not by subsequent treatment of the same brains with chloroquine (100 μM) (F(4,10)=70.47, *P*<0.001; reserpine 1 μM+chloroquine 100 μM, *P*<0.001), demonstrating that functional VMAT is necessary for amphetamine-induced vesicular alkalization. dVMAT-pHluorin intensity changes were measured at *λ*_ex_=920 nm, *λ*_em_=525/50 nm FWHM. Pooled concentration-response data for **b**–**d** show fluorescence changes normalized to that evoked by subsequent chloroquine (100 μM) treatment in each brain, and those in **e** were normalized to initial fluorescence intensity. Drugs were sequentially applied to the brain preparation by bath superfusion. Fluorescence was measured before treatment and after a 10 min drug equilibration period (25 °C). Data are represented as mean fluorescence intensities±s.e.m. in the MB-MV1 region from *N*⩾3 experiments.

We expressed the dVMAT-pHluorin biosensor in presynaptic dopamine nerve terminals to directly monitor amphetamine-induced pH changes in dopamine synaptic vesicles in a whole brain for the first time. As a standard for comparison, we used chloroquine (100 μM) to disrupt the vesicular H^+^ gradient and thus to alkalize the lumen relative to the acidic starting pH. Amphetamine (1 μM) also caused significant concentration-dependent alkalization, that is, deacidification, of the vesicle (F(3, 16)=29.96, *P*<0.001; Fig. 5a,b). This amphetamine-induced rise in vesicle pH was sustained during continuous application of the drug by bath superfusion (Supplementary Fig. 7a). Methamphetamine (10 μM) also caused significant vesicular alkalization (F(3, 20)=9.03, *P*=0.001; Supplementary Fig. 8a). In contrast, treatment with the psychostimulant methylphenidate, a non-substrate DAT inhibitor^21^ that is also a lipophilic weak base (calculated pK_a_=9.5 with Log*D*=0.24, pH 7.4 (ref. 35)), did not lead to intraluminal vesicular pH changes (*P*>0.05) (Fig. 5c) even at 100 μM, a concentration far higher than that needed to block DAT^36^,^37^.

By expressing dVMAT-pHluorin in the *Drosophila DAT* (*dDAT*) null *fumin* mutant background^38^, we were able to examine the role of dDAT in transporting amphetamines into cells by using vesicle alkalization as a readout. Specifically, we asked whether passive diffusion of amphetamines across the plasma membrane supplies sufficient intracellular amphetamine to produce vesicular alkalization or whether it must be imported by the concentrative transporter dDAT. We found that in the absence of functional dDAT, amphetamine did not significantly raise vesicle pH even at concentrations up to 700-fold greater than the 1 μM concentration that caused significant vesicle alkalization in the WT background (*P*>0.05; Fig. 5d). Methamphetamine-induced vesicle alkalization was also blocked in the *dDAT* null mutant background (Supplementary Fig. 8b). In contrast, subsequent treatment of the same *dDAT* null brains with chloroquine (100 μM) still produced significant vesicle alkalization (Fig. 5d, Supplementary Fig. 8b). This is consistent with chloroquine's ability to lipophilically diffuse across membranes and also demonstrates that the synaptic vesicles in *dDAT* null brains are capable of intraluminal alkalization.

Next, we used a pharmacological strategy to block dVMAT to determine whether its function at the vesicle membrane is necessary for amphetamine-induced vesicle alkalization. Pretreatment with the inhibitor reserpine (1 μM; dVMAT IC_50_=258 nM (ref. 39)) prevented amphetamine- and methamphetamine-induced vesicle alkalization (Fig. 5e, Supplementary Fig. 8c), even with extended incubations of increasing amphetamine concentrations (100 nM–10 μM; Supplementary Fig. 7a). Similarly, the VMAT inhibitor (+)-CYY477 blocked amphetamine-induced synaptic vesicle alkalization (Supplementary Fig. 9). In contrast, subsequent alkalization by chloroquine in the same brains (*P*<0.001) was unaffected by reserpine blockade of VMAT (Fig. 5e, Supplementary Figs 7b and 8c), highlighting the ability of chloroquine to diffuse across the vesicle membrane independently of VMAT, just as it diffuses across the plasma membrane independently of DAT (Fig. 5d).

### Substrate-coupled H^+^ antiport by VMAT alkalizes vesicles

**Figure 6:**
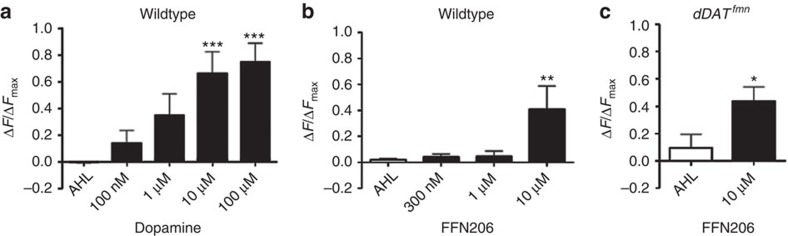
VMAT substrates dopamine and FFN206 alkalize synaptic vesicles in dopamine nerve terminals. (**a**) Dopamine caused concentration-dependent alkalization as indicated by dVMAT-pHluorin brightening (F(5, 21)=6.74, *P*<0.001; 10 μM dopamine, *P*=0.006; 100 μM dopamine, *P*=0.002). (**b**) FFN206 significantly alkalized synaptic vesicles at 10 μM (F(3, 12)=4.10, *P*=0.032; 10 μM FFN206, *P*=0.03). (**c**) In the *dDAT* null background flies, FFN206 produced brightening of dVMAT-pHluorin (*P*=0.035), indicating that it does not require functional DAT to attain a sufficient concentration in vesicles. Increases in fluorescence in MB-MV1 regions (compared with vehicle, AHL) after 10-min drug treatment were normalized to final chloroquine (100 μM) changes (Δ*F*_max_). Drugs were sequentially applied to the brain preparation by bath superfusion. Fluorescence was measured before treatment and after a 10-min drug equilibration period (25 °C). Data are represented as mean intensities±s.e.m. in the MB-MV1 region from *N*>3 experiments.

The experiments above clearly demonstrated that amphetamines cause vesicle alkalization in dopamine terminals in an *ex vivo* whole-brain preparation. At low micromolar concentrations of amphetamines, this alkalization requires concentrative transporters at both the plasma membrane (DAT) and the synaptic vesicle membrane (VMAT). Notably, VMAT functions by stoichiometrically exporting 2H^+^ from the vesicle lumen for each cationic monoamine substrate it imports^40^. We hypothesized that if amphetamines are truly VMAT substrates, then their transport should diminish the vesicular ΔpH through the substrate-coupled H^+^antiport mechanism. We therefore tested whether proven VMAT substrates also alkalize vesicles. The endogenous substrate dopamine caused significant concentration-dependent vesicular alkalization (F(5, 21)=6.74, *P*<0.001; Fig. 6a), like amphetamine and methamphetamine. Similarly, FFN206, which is also a reserpine-sensitive VMAT substrate^17^ (Fig. 3), led to significant vesicle alkalization at 10 μM (F(3,12)=4.10, *P*=0.032; Fig. 6b). However, unlike amphetamine or methamphetamine, 10 μM FFN206 still alkalized vesicles in presynaptic nerve terminals in the *dDAT* null mutant expressing dVMAT-pHluorin, demonstrating that FFN206 requires dVMAT but not dDAT for vesicle entry (Figs 3b,e,f and 6c). Notably, significant vesicular pH changes were not observed with FFN206 at 1 μM or at the 300 nM concentration we used as a monoamine tracer (Fig. 6b).

**Figure 7:**
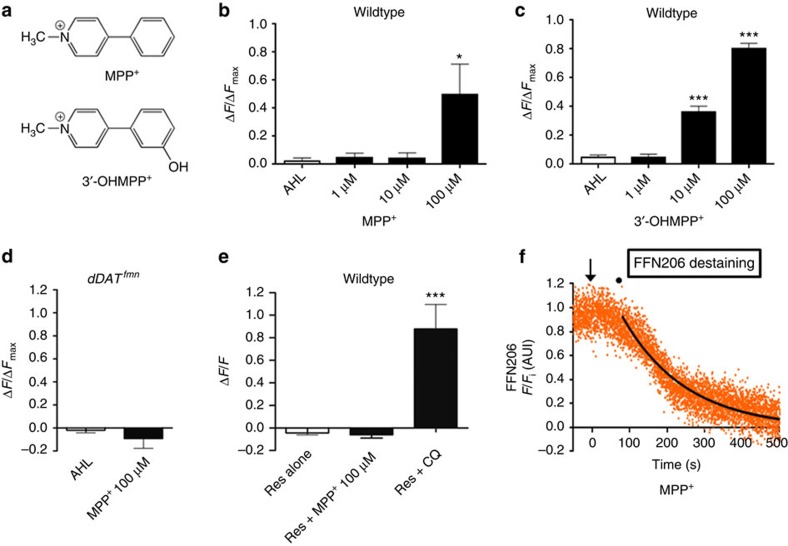
MPP^+^ and 3′-OHMPP^+^ alkalize synaptic vesicles and MPP^+^ discharges FFN206 from vesicles in dopamine nerve terminals. (**a**) Structural formulae of MPP^+^ and 3′-OHMPP^+^. (**b**) MPP^+^ (100 μM) significantly alkalized synaptic vesicles as indicated by dVMAT-pHluorin brightening (F(3,16)=137.93, *P*<0.001). (**c**) Escalating doses of 3′-OHMPP^+^ significantly raised vesicle intraluminal pH (*P*<0.001) in a concentration-dependent manner. 3′-OHMPP^+^ was effective at a 10-fold lower concentration than MPP^+^. (**d**) In *dDAT* null mutants, MPP^+^ (100 μM) did not significantly raise synaptic vesicle pH (*P*>0.05). (**e**) Reserpine (1 μM) blocked MPP^+^ (100 μM) but not chloroquine (100 μM)-induced vesicle alkalization (F(2, 12)=18.02, *P*<0.001; 1 μM reserpine+100 μM MPP^+^, *P*>0.05; 1 μM reserpine+100 μM chloroquine, *P*<0.001). (**f**) MPP^+^ (100 μM) caused FFN206 destaining in presynaptic dopamine nerve terminals of adult TH Rescue brain. A representative plot was best fit to an initial plateau followed by a monoexponential decay (black; for *N*=4 experiments, *t*_1/2_=83.96±15.98 s; *R*^2^=0.96). Arrow indicates drug addition and circle represents the point at which 50% of drug is in the imaging chamber. (**b**–**d**) Mean change in fluorescence intensities±s.e.m. in the MB-MV1 region from *n*⩾3 experiments, normalized to fluorescence evoked by subsequent chloroquine (100 μM) treatment in the same brains. (**e**) Mean change in fluorescence intensities±s.e.m. in the MB-MV1 region from *n*⩾3 experiments, normalized to initial fluorescence. (**b**–**e**) Drugs were sequentially applied to the brain preparation by bath superfusion. Fluorescence was measured before treatment and after a 10-min drug equilibration period (25 °C). (**f**) Fluorescence decay of MB-MV1 region normalized to fluorescence after steady-state loading with FFN206 (300 nM).

That amphetamine and methamphetamine alkalize vesicles with similar concentration dependence as established VMAT substrates, and even more importantly, that alkalization by amphetamines can be blocked by VMAT inhibitors, together support our hypothesis that substrate-coupled H^+^-antiport accounts for the observed alkalization. Nevertheless, given that amphetamine, methamphetamine, dopamine, and FFN206 are weak bases, it is conceivable that if they were transported in the neutral state, then the neutral species could bind protons from the acidic vesicle lumen, eventually dissipating the vesicular ΔpH. To determine whether substrate-coupled H^+^-antiport by VMAT is sufficient to alkalize vesicles, it was necessary to test a VMAT substrate that is not a weak base. Thus, we used the known DAT and VMAT substrate, 1-methyl-4-phenylpyridinium (MPP^+^), which cannot buffer luminal H^+^ due to the fixed positive charge of its methyl-pyridinium nitrogen^41^,^42^ (Fig. 7a).

### MPP^+^ confirms the mechanism of vesicle alkalization by VMAT

Bath application of 100 μM MPP^+^ significantly alkalized synaptic vesicles in presynaptic dopamine nerve terminals (*P*=0.028; Fig. 7b). In contrast, there was no significant dVMAT-pHluorin brightening in presynaptic dopamine nerve terminals following MPP^+^ treatment in either the *dDAT* null mutant (Fig. 7d) or in WT brains pretreated with reserpine (*P*>0.05; Fig. 7e), consistent with MPP^+^'s dependence on both dDAT and dVMAT. Moreover, MPP^+^ at the concentration that produced dVMAT-pHluorin brightening (100 μM, Fig. 7b) also destained FFN206 in dopamine terminals of TH Rescue brains even in the continuous presence of FFN206. Destaining showed monoexponential kinetics similar to amphetamine (*t*_1/2_=83.96±15.98 s, R^2^=0.96±0.01; Fig. 7f), suggesting that, like amphetamines, MPP^+^ can also cause redistribution of dopaminergic vesicle contents through vesicle alkalization.

The relatively low potency of MPP^+^ compared with amphetamine for vesicular alkalization and FFN206 destaining is consistent with its low affinity for VMAT2 (*K*_i_=92±14 μM) (ref. 43). Therefore, we also tested 3′-OHMPP^+^, a MPP^+^ derivative with a higher affinity for VMAT2 (*K*_i_=2.4±0.1 μM) (ref. 43). Indeed, 3′-OHMPP^+^ caused significant vesicular alkalization (*P*<0.001) at concentrations 10-fold lower (10 μM) than MPP^+^ (compare Fig. 7b,c). Thus, despite the fact that they are not weak bases, MPP^+^ and 3′-OHMPP^+^ can still alkalize the vesicle lumen like amphetamine, methamphetamine, dopamine, and FFN206, which are weak bases. Critically, the property shared by these diverse compounds is their ability to be transported by VMAT. This finding indicates that transport-dependent H^+^ antiport by VMAT is sufficient to diminish intraluminal H^+^ concentration and alkalize the vesicle lumen without a need for H^+^ buffering.

**Figure 8:**
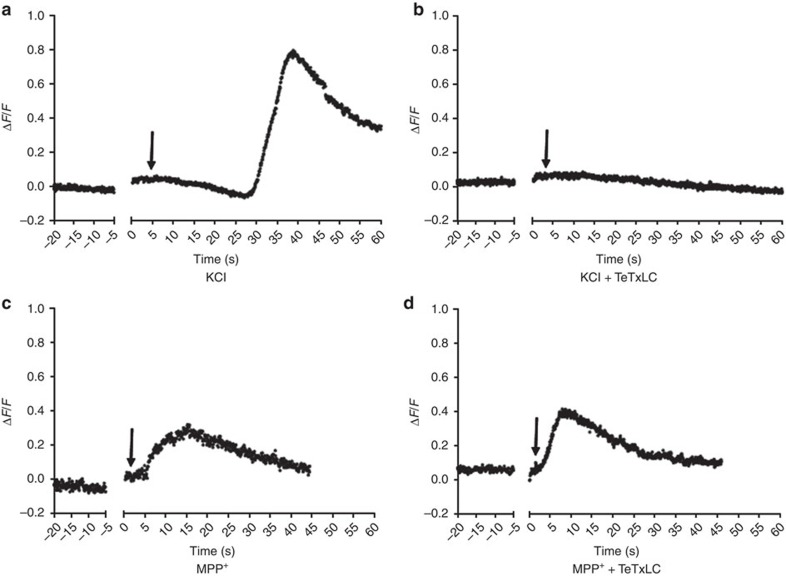
MPP^+^-induced dopamine synaptic vesicle alkalization in dopamine nerve terminals is reversible and independent of exocytosis. (**a**) Pressure ejection of KCl (10 s, arrow) onto an *ex vivo* WT whole fly brain preparation led to a delayed brightening of TH-driven dVMAT-pHluorin with a slow decay. (**b**) The fluorescence response to KCl application (10 s, arrow) was abolished in fly brains expressing both dVMAT-pHluorin and tetanus toxin light chain (TeTxLC), a known blocker of exocytosis, in dopamine nerve terminals. (**c**) MPP^+^ application (1 s, arrow) caused a more rapid dVMAT-pHluorin brightening in WT brain with a rapid return to baseline. (**d**) TeTxLC co-expression with dVMAT-pHluorin did not, however, diminish the transient fluorescence response to MPP^+^ application (1 s). All traces show single-plane fluorescence intensity measured at 100-ms intervals, integrated over the MB-MV1 region, and normalized to initial values. Each trace shows 20 s of extended baseline prior to drug application and is representative of *N*⩾3 experiments.

This proposed antiport mechanism for substrate-induced vesicular alkalization is not dependent on vesicle exocytosis. An alternative explanation for dVMAT-pHluorin brightening could be that vesicle exocytosis shifts the sensor from the acidic vesicle lumen to the neutral extracellular milieu. To differentiate these mechanisms, we again took advantage of MPP^+^'s properties as a non-protonatable VMAT substrate that cannot buffer H^+^. KCl or MPP^+^ were applied to brains expressing TH-driven dVMAT-pHluorin as well as a genetically encoded Tetanus toxin light chain (TeTxLC), a known blocker of exocytosis^44^. Given the relatively fast kinetics of exocytosis, images were acquired at higher frequency to detect rapid vesicular pH changes. Brief pressure ejection of KCl led to a prolonged, intense pH change (Fig. 8a) that was abrogated in flies expressing TeTxLC (Fig. 8b). In contrast, brief application of MPP^+^ caused rapid, transient intraluminal alkalization (Fig. 8c) that was not affected by TeTxLC expression (Fig. 8d). We also used the same genetic background of TeTxLC co-expression with dVMAT-pHluorin to construct a vesicle intraluminal pH calibration curve using a cocktail of ionophores across a broad pH range (see Methods); the TeTxLC was required to avoid the potential confound of vesicle exocytosis during calibration. By interpolating from this curve, we determined that the mean peak change in fluorescence intensity induced by transient application of MPP^+^ in these TeTxLC experiments (Δ*F*/*F*=0.31±0.07) corresponded to a 0.4 pH unit change from the baseline pH of ∼5.8 (95% confidence interval: 5.48–6.08) (Supplementary Fig. 6c). These results confirmed that MPP^+^ changes vesicle pH by a mechanism independent of both exocytosis and H^+^ buffering but dependent on VMAT. Taken together, our results point to the intrinsic substrate:H^+^ antiport function of VMAT as the mechanism by which amphetamines, or, more generally, any known VMAT substrate, can elevate the pH within intact synaptic vesicles if it is present in the cytoplasm at an adequate concentration.

## Discussion

Although there is consensus that amphetamines produce behavioural effects by raising extracellular dopamine levels, diverse and often contradictory inferences have been drawn to explain the molecular basis of this effect. In order to reveal amphetamine's actions at the synaptic vesicle level, we have combined a number of novel, complementary genetic, optical and pharmacological approaches to address these questions.

In both locomotor and self-administration behavioural paradigms in rodents, acute preadministration of the novel VMAT2 inhibitor (+)-CYY477 abolished the effects of amphetamine and methamphetamine, indicating that VMAT2 function is obligatory for amphetamines to produce their acute behavioural effects. These data are consistent with work by Dwoskin and colleagues who have developed inhibitors of methamphetamine behavioural actions using lobeline analogues. During their structure-driven pre-clinical studies, this group recognized that the drugs shared the common property of being VMAT blockers^45^. Comparable behavioural effects against methamphetamine were also observed with the VMAT2 blocker tetrabenazine, but this drug shows only modest selectivity for VMAT2 over DAT^46^. We tried to improve on the selectivity and duration of action of tetrabenazine by synthesizing (+)-CYY477, a derivative of the high-affinity analogue dihydrotetrabenazine. We found that (+)-CYY477 indeed has high VMAT2 affinity but negligible affinity for DAT (Supplementary Table 1). Selectivity for VMAT2 over DAT is especially important for discriminating between these transporters as sites of action for amphetamines, which act at both. Taken together, our data demonstrating (+)-CYY477's ability to block vesicular dopamine uptake *in vitro* and to induce catalepsy at high doses *in vivo* are consistent with other established VMAT inhibitors like tetrabenazine and suggest that, like its parent compound, (+)-CYY477 is a potent and effective VMAT2 blocker.

The ability of (+)-CYY477 and other VMAT inhibitors^46^,^47^,^48^ to block amphetamines' behavioural actions rules out two widely touted mechanisms for the *in vivo* action of these psychostimulants at the doses we studied: (1) It has been proposed that amphetamines elevate cytoplasmic dopamine by blocking sequestration of endogenous dopamine into vesicles by VMAT^7^, thus making more dopamine available for efflux by DAT. If this were the case, then VMAT inhibitors would mimic or enhance the behavioural effects of amphetamines. Instead, we found that (+)-CYY477 antagonized the effects of amphetamines. (2) It has been proposed that, like cocaine, amphetamines elevate extracellular dopamine by competing for dopamine uptake by DAT^49^,^50^. If this were so at pharmacologically relevant amphetamine concentrations, then the selective VMAT2 inhibitor (+)-CYY477, which has negligible affinity for DAT, should not abolish the acute actions of amphetamines at DAT. Instead, we found that (+)-CYY477 antagonized the effects of amphetamines, although it did indeed spare cocaine, consistent with cocaine's competition with dopamine at DAT. That cocaine-induced behaviours are spared by (+)-CYY477 also indicates that dopamine stores and neurotransmission remain functionally intact after acute pretreatment with the VMAT2 inhibitor.

The profound and selective antagonism of amphetamine-induced behaviours by (+)-CYY477 points to a critical role for VMAT function in the acute actions of amphetamines upstream of, and in addition to, their established action to facilitate efflux of cytoplasmic dopamine through DAT.

We used a fly model system to elucidate the mechanism of amphetamines' actions at synaptic vesicles *in vivo*. We recently introduced FFNs to enable rapid imaging of monoamine storage and release dynamics in vertebrate brain slice^30^,^51^. A new FFN, FFN206 (ref. 17) has proved to be an excellent marker of monoaminergic nerve terminals in fly brain. To focus specifically on dopaminergic terminals, we used flies genetically engineered to express dVMAT only in TH-expressing dopamine neurons. FFN206 labelling was reserpine-sensitive and was dissipated by KCl-induced depolarization, consistent with the accumulation of FFN206 in synaptic vesicles by dVMAT. Thus, this probe serves as a sensitive and selective surrogate marker for dopamine content in small synaptic and large dense core vesicles^52^. Treatment with 1 μM amphetamine diminished FFN206 signal to levels comparable to surrounding neuropil background. While similar kinetics of amphetamine-induced destaining of previous generation FFN molecules loaded into brain slices has been shown^51^, this is the first report of amphetamine-induced dopamine vesicle content release in whole brain.

Since vesicular pH plays an important role in storage and release of vesicle contents, we used the recently developed vesicular pH biosensor, dVMAT-pHluorin, as a tool to elucidate amphetamines' effects on intraluminal pH within intact vesicles in our whole-brain preparation. This approach differs from earlier uses of pHluorin biosensors to monitor vesicle dynamics during exo- and endocytosis^31^,^53^. With dVMAT-pHluorin we observed that a clinically relevant amphetamine concentration (1 μM) alkalized dopamine vesicles within minutes in a VMAT-dependent manner.

The concentrations of amphetamines that alkalize fly brain dopaminergic vesicles and discharge their content are well within the range of behaviourally active amphetamine or methamphetamine levels found in human and rodent plasma (∼0.7 μM) (refs 54, 55) and close to target plasma levels of *S*(+)amphetamine in therapeutic treatment of children with attention deficit hyperactivity disorder (0.5–1.1 μM) (ref. 56) as well as in chronic methamphetamine users (1–3 μM) (ref. 57). Similarly, in rodents and non-human primates, behaviourally active doses of amphetamines yield brain concentrations (1–10 μM: striatal microdialysate)^57^,^58^,^59^,^60^ that are in the range of those causing VMAT-dependent vesicular alkalization in fly brain, although lower concentrations have also been reported^50^.

Lipophilic weak bases like chloroquine cause vesicle alkalization and readily redistribute vesicle contents as we showed in whole brain with FFN206. However, two lines of evidence demonstrate that, at relevant concentrations, amphetamines do not work by the same mechanisms as chloroquine. (1) Our data show that disruption of DAT or VMAT function through genetic or pharmacological manipulation profoundly reduced amphetamine's potency to alkalize the vesicle lumen. The requirement for functional transporters at both the plasma membrane and the vesicular membrane for amphetamine action challenges the common assumption that lipophilic diffusion alone can deliver enough amphetamine to vesicles to alkalize vesicles, even at high micromolar concentrations of the drug. By contrast, neither deletion of DAT nor blockade of VMAT hindered chloroquine-induced alkalization. (2) That FFN206 is accumulated in puncta in a VMAT-dependent manner gives optical proof that mildly lipophilic compounds can be concentrated and that the vesicular membrane provides a sufficient barrier to their egress. Since amphetamine (Log*D*=−0.79 at pH 7.4 (ref. 15)) is less lipophilic than FFN206 (Log*D*=−0.54 at pH 7.4 (ref. 17)), it should be even less capable of passive diffusion into or out of vesicles. Altogether, these data provide the most direct evidence to date that the amphetamines, like dopamine and FFN206, are *bona fide* substrates of VMAT and not merely inhibitors.

We next aimed to distinguish between two possible mechanisms for this alkalization: (1) Alkalization of the acidic lumen could occur by net export of 2H^+^ via VMAT for every amphetamine molecule transported into the vesicle (substrate:2H^+^ antiport)^61^ or (2) substrates including amphetamines that are weak bases could buffer luminal H^+^ following their VMAT-dependent transport into the lumen if their neutral, unprotonated species were transported^2^. Since protonated amphetamine species (pK_a_=9.9) outnumbers neutral species by ∼300:1 in the cytosol at physiological pH, it seems unlikely that sufficient neutral species would be transported into the vesicle lumen to significantly buffer the acidic environment^15^. Instead, our data are more consistent with the cationic substrate:H^+^ antiport mechanism, especially since the permanently charged VMAT substrates MPP^+^ and 3′-OHMPP^+^ also alkalize vesicles even though they are not weak bases. Thus, we infer that the H^+^ antiport-driven process of substrate translocation by VMAT is sufficient to account for the vesicular alkalization produced not only by MPP^+^ and 3′-OHMPP^+^, which cannot be protonated, but also by the VMAT substrates dopamine, amphetamine, methamphetamine and FFN206, which have basic amino groups that are predominantly protonated at physiological pH.

Measurements of synaptic vesicle pH are typically made in cells or in isolated vesicles, and to our knowledge this study represents the first attempt to measure the intraluminal pH of synaptic vesicles in a whole living brain. Ionic manipulations used for calibrating pH measurements can lead to exocytosis, confounding interpretation of the data. To prevent exocytosis, we used flies with the TeTxLC genetic background to generate a pH calibration curve and found the baseline intraluminal pH of dopamine vesicles in the fly brain to be ∼5.8, comparable to that reported for mammalian synaptic vesicles (pH 5.7) (ref. 62). This value is substantially more acidic than reported by Sturman *et al*.^63^ (pH 6.6) for synaptic vesicles in the fly neuromuscular junction. This disparity could be due to differences in genetic backgrounds, biosensors, sites of measurement and/or methods of calibration.

Given that the vesicular V-ATPase is the principal driver for maintaining the acidic intraluminal pH and H^+^ electrochemical gradient (ΔpH), we propose that net vesicular alkalization occurs if the rate of VMAT-mediated H^+^ efflux exceeds the rate of V-ATPase-mediated H^+^ import. The rapid and reversible alkalization induced by transient MPP^+^ pressure ejection highlights the dynamic interplay of these opposing processes within intact vesicles. We believe that this alkalization results from elevated cytoplasmic pools of substrates driving VMAT-mediated substrate:H^+^ antiport at a sufficient rate to temporarily outstrip the ability of the V-ATPase to maintain the original acidic vesicular pH.

Our data lead to a model for how pharmacologically relevant concentrations of amphetamines increase extracellular dopamine: (1) DAT imports and concentrates amphetamines in the cytoplasm. (2) Cytoplasmic amphetamines (and endogenous dopamine) are accumulated into vesicles by VMAT in a H^+^-antiport process that diminishes vesicular H^+^ concentration. (3) Diminished vesicular ΔpH promotes dopamine redistribution from vesicles into the cytoplasm through a mechanism that is still unclear. Vesicle deacidification alters the protonation state of luminal dopamine, which might be sufficient to increase its diffusion across the membrane. However, this mechanism does not readily explain protonophore-induced efflux of MPP^+^ from vesicles^64^, since this compound is a quaternary ammonium and thus pH cannot alter MPP^+^'s protonation state. Whether VMAT itself might be a route of dopamine release from vesicles^11^,^65^ requires further study. (4) The redistributed cytoplasmic dopamine subsequently effluxes out of the cell through DAT via amphetamine-stimulated reverse transport. Our previous work demonstrated that phosphorylation of DAT is essential for dopamine efflux^66^ and for locomotor behaviour induced by amphetamine but not for the actions of methylphenidate, a competitive non-substrate inhibitor of DAT^20^,^21^. These results are consistent with the inference from our rodent behavioural data that competitive inhibition at DAT by amphetamines is not sufficient to produce behavioural effects at the concentrations tested. By the same logic, stimulation of dopamine synthesis by TH and/or inhibition of dopamine catabolism by monoamine oxidases, both of which can elevate cytoplasmic dopamine concentrations^6^, are insufficient in themselves to produce amphetamines' acute behavioural effects. These data highlight the functional coupling of DAT and VMAT as critical to amphetamines' actions *in vivo*. Because amphetamines are also substrates of norepinephrine transporter and serotonin transporter, and VMAT is present in adrenergic and serotonergic neurons^6^, the tandem actions of plasma membrane transporters and VMAT are likely important for amphetamine-induced release of other monoamines as well. Furthermore, our results demonstrate the first application of a novel experimental system that can be used to develop important new insights into the physiology of intact monoaminergic vesicles.

## Methods

### Fly strains

*D. melanogaster* strains include the WT strain, w^1118^CS_10_ (wCS10), which is w^1118^ outcrossed into Canton-S for 10 generations^68^. The *dDAT* null *fumin* mutant *dDAT*^*fmn*^ (gift of K. Kume, Kumamoto University)^38^, *dVMAT* null *dVMAT1*^*P1*^^69^ and *UAS-dVMAT* transgene corresponding to the neuron-specific dVMAT-A isoform have been previously described^38^,^68^,^69^. GAL4 expression driver lines include TH-GAL4 (a gift of Dr S. Birman, Université Aix-Marseille II-III)^22^ and Tdc2-GAL4^24^ which were previously shown to drive expression in DA and TA/OA neurons, respectively^22^,^24^. To detect pH changes in the intravesicular lumen of monoaminergic synaptic vesicles, we constructed a new fly strain with the novel transgene, *UAS-dVMAT-pHluorin* in both WT *dDAT* and *dDAT*^*fmn*^ genetic backgrounds. All fly strains were grown and maintained on standard cornmeal-molasses media at 25 °C under a 12-h light–dark schedule.

### Construction of transgenic fly strains

The dVMAT-pHluorin fly strain was created by injection of the dVMAT-pHluorin probe sequence into WT fly embryos. The probe was generated by insertion of the pH-sensitive, super-ecliptic pHluorin DNA into the first luminal loop of dVMAT-A and surrounded by 5′ linker YPYDVPGSTSGGSGGTGG and 3′ linker SGGTGGSGGTGGSGGTGYAT between Arg-182 and Pro-183 of dVMAT-A. dVMAT-pHluorin was subsequently genetically recombined with the TH-GAL4 expression driver on chromosome III. This allowed us to achieve improved probe expression in the resulting homozygous fly strain compared with the initial description of the probe^18^.

dVMAT rescue fly strains were constructed by introducing the *UAS-dVMAT* transgene into the *dVMAT*^*P1*^ null genetic background to selectively rescue dVMAT function in DA and OA/TA neurons using the TH-GAL4 and Tdc2-GAL4 expression drivers, respectively. The following genotypes were described in the text: (1) ‘TH Rescue': *w*^*−*^*; dVMAT*^*P1*^; *TH-GAL4*, *UAS-dVMAT* and (2) ‘Tdc Rescue': *w*^*−*^; *dVMAT*^*P1*^, *Tdc2-GAL4*; *UAS-dVMAT*. To examine dVMAT-pHluorin's vesicular localization, we expressed the *UAS-dVMAT-pHluorin* transgene driven by the elav-GAL4 expression driver: *elav-GAL4;;UAS-dVMAT-pHluorin*. To test the effects of the respective expression drivers alone in the *dVMAT* null background, we used the following fly strains: (1) *w*^*−*^; *dVMAT*^*P1*^; *TH-GAL4* and (2) *w*^*−*^; *dVMAT*^*P1*^, *Tdc2-GAL4*. Fly strains including the *UAS-dVMAT* transgene and *dVMAT*^*P1*^ allele were also outcrossed for 10 generations into the wCS10 WT genetic background. To test whether dVMAT-pHluorin itself is functional *in vivo*, we expressed the *UAS-dVMAT-pHluorin* transgene driven by the Tdc2-GAL4 expression driver in the *dVMAT* null background using the *w*^*−*^; *dVMAT*^*P1*^, *Tdc2-GAL4*; *UAS-dVMAT-pHluorin* fly strain, eliminating the potential confound of endogenous dVMAT expression.

### Fly brain imaging

*Drug treatments*. All drugs were diluted in adult haemolymph-like saline (AHL; 108 mM NaCl, 5 mM KCl, 2 mM CaCl_2_, 8.2 mM MgCl_2_, 1 mM NaH_2_PO_4_, 10 mM sucrose, 5 mM trehalose, 5 mM HEPES, 4 mM NaHCO_3_; pH 7.5, 265 mOsm). In most experiments, drugs were applied by bath superfusion into a flow chamber at room temperature (25 °C). Fluorescence was typically measured before treatment and after a 10 min drug equilibration period (25 °C). In some experiments, drugs were also applied by air pressure ejection (0.1–10 s) duration onto brains submerged in AHL as described earlier^16^. Drug was delivered to a pulled glass pipet directed at the MB-MV1 region at a distance of ∼10 μm away from the brain using Picospritzer II (Parker Hannifin Corporation, Cleveland, OH). To examine FFN206-labelling of presynaptic DA nerve terminals, we continuously superfused FFN206 (300 nM, 30 min) and examined brain neuropil by multiphoton laser scanning microscopy. To induce synaptic vesicle exocytosis or alkalization, we treated brains with KCl (40 mM, in AHL adjusted for ionic osmolarity) or CQ (100 μM) solutions, respectively. In experiments measuring destaining of FFN206 from TH Rescue brains, the brains were loaded to steady state with 300 nM FFN206 and then treated with drug solutions also containing 300 nM FFN206. To inhibit dVMAT activity, we pretreated brains with either reserpine or (+)-CYY477 (1 μM, 10–20 min) and imaged in the continuous presence of the respective blocker. To test effects on vesicular pH, brains were incubated with 0.1–700 μM amphetamine, methamphetamine, MPH, DA, FFN206, MPP^+^ or 3′-OHMPP^+^ in fly brains expressing dVMAT-pHluorin in presynaptic DA neurons in either a WT *dDAT* or *dDAT*^*fmn*^ genetic background. In experiments using DA, we pretreated brains with monoamine oxidase (MAO) inhibitors pargyline and selegiline (10 μM, 10 min).

*Imaging*. An isolated, *ex vivo* whole adult fly brain preparation was obtained by rapid removal and microdissection of the brain from decapitated flies as previously described^16^. A significant advantage of this preparation is that following removal of head cuticle and connective tissues, drugs are applied directly to brain tissue at known concentrations. This whole-brain preparation was imaged with continuous flow on an Ultima multiphoton laser scanning microscope (Prairie Technologies Bruker Corp., Middleton, WI). Fluorescent emission was collected using a 460 nm/50 nm FWHM bandpass emission filter for FFN206 and 525/50 nm FWHM bandpass filter for dVMAT-pHluorin.

### Compounds

The drugs used in the present study including their respective salt and enantiomeric forms were as follows and purchased from Sigma-Aldrich (St Louis, MO) unless indicated otherwise: D-amphetamine hemisulphate, D-methamphetamine HCl, cocaine HCl (Merck, Whitehouse Station, NJ), methylphenidate HCl (MPH), bafilomycin A1 (Santa Cruz Biotechnology, Dallas, TX), 3-hydroxytyramine HCl (dopamine), 2-(*N*-morpholino)ethanesulfonic acid (MES), 1-methyl-4-phenylpyridinium iodide (MPP^+^), carbonyl cyanide 4-(trifluoromethoxy)phenylhydrazone (FCCP), monensin A sodium salt (monensin), nigericin sodium salt (nigericin), *N*-methyl-*N*-propargylbenzylamine hydrochloride (pargyline HCl), reserpine, R(−)-*N*-α-dimethyl-*N*-2-propynyl-benzeneethanamine hydrochloride (selegiline HCl), *N*-Methyl-*N*-propargyl-3-(2,4-dichlorophenoxy)propylamine hydrochloride (clorgyline HCl), 2-Cyano-*N*,*N*-diethyl-3-(3,4-dihydroxy-5-nitrophenyl)propenamide (entacapone), chloroquine diphosphate (CQ), (±)-tetrabenazine (TBZ), haloperidol, Ro4-1248, tetrodotoxin (Tocris Bioscience, Ellisville, MO), D-tubocurarine chloride (Tocris Bioscience), Triton X-100, digitonin (Santa Cruz Biotechnology), valinomycin, adenosine 5′-triphosphate magnesium salt (ATP), L-ascorbic acid, potassium sodium tartrate tetrahydrate, bovine serum albumin (fraction V; EMD Millipore, Billerica, MA), green food coloring dye (La Flor Products, Ridgewood, NY). [^3^H]dihydrotetrabenazine, [^3^H]nisoxetine, [^3^H]citalopram and [^3^H]dopamine were obtained from American Radiolabelled Chemicals (St Louis, MO), and [^3^H]WIN 35,428 was obtained from PerkinElmer Life Sciences (Waltham, MA). FFN206 (synthesized in the Department of Chemistry, Columbia University, New York, NY), 1-methyl-4-(3′-hydroxyphenyl)phenylpyridinium iodide (3′-OHMPP^+^; synthesized in the Dept. of Chemistry, Wichita State University, Wichita, KS) and (+)trans-10-desmethyltetrabenazine [(+)-CYY477] (synthesized at the School of Pharmacy, College of Medicine, National Taiwan University, Taipei, Taiwan).

### Preparation of (**+**)-CYY477

Preparation of (+)-CYY477 is described in detail in the Supplementary Information. Briefly, treatment of commercially available 7-hydroxy-6-methoxy-3,4-dihydroisoquinoline with 2-acetyl-*N,N,N*,4-tetramethylpentan-1-aminium iodide salt in refluxing ethanol provided racemic *trans*-10-desmethyltetrabenazine [(±)-CYY477] in 59% yield. Optical resolution of (±)-CYY477 using (+)-2,3-dibenzoyl tartaric acid ((+)-DBT) in ethanol yielded optically pure (+)-CYY477 (*ee*>99%) according to our modifications to previously described methods^70^ (see Supplementary Note 1, Supplementary Figs 10–13).

### Assay of Dopamine Uptake by VMAT2

293-rV2 cells, HEK293 cells stably transfected with rat VMAT2, were obtained from Dr R.H. Edwards and have been previously characterized by Adam *et al*. (2008) and by Hu *et al*.^17^,^71^_ENREF_68. Cells were grown in DMEM in 10% fetal bovine serum, 100 units per ml penicillin, 100 μg ml^−1^ streptomycin (37 °C, 5% CO_2_) in polystyrene plates to 85% confluency. On the day of assay, confluent cells were first washed with PBS, trypsinized, and washed in DMEM before being placed in Resuspension Buffer (110 mM L(+)-K-Na-tartaric acid, 5 mM MgCl_2_, 5 mM D-glucose, 20 mM HEPES-K, pH 7.4) at 37 °C. Cells were counted and resuspended at a concentration of ∼100,000 cells per 250 μl in Assay Buffer (Resuspension Buffer supplemented with 5 mM Mg-ATP, 1 mM ascorbate, 0.2% bovine serum albumin, 10 μM digitonin, 10 μM of the COMT inhibitor entacapone and 10 μM each of the MAO inhibitors selegiline, clorgyline, and pargyline). 250 μl aliquots of cells were preincubated (10 min, 37 °C) either with 25 μl of the respective test compound in Assay Buffer or with 25 μl of buffer without compound. Test compounds were dissolved in DMSO, which was always <0.1% final concentration in the assay; digitonin was dissolved in 100% ethanol (0.1% final concentration). Thereafter, 25 μl of [^3^H]dopamine (60 Ci mmol^−1^) suspended in Assay Buffer was added to the assay mixture at a final concentration of approximately 10 nM (300 μl final volume). After incubation with [^3^H]dopamine (10 min, 37 °C ), the assay mixture was diluted to 1.5 ml with ice cold Wash Buffer (85 mM NaCl, 85 mM KCl, 5 mM MgCl_2_, 20 mM HEPES-K, pH 7.4) and immediately vacuum-filtred with a Brandel 24 well manifold (Brandel Instruments) and captured on prewetted Whatman GF/B filters. The filtres were rapidly washed twice with 1.5 ml Wash Buffer and then dissolved in Safety Solve Complete Counting Cocktail liquid scintillant (Research Products International, Mt. Prospect, IL) and counted on a Packard Tri-Carb 2100 TR liquid scintillation analyzer (PerkinElmer, Hopkinton, MA) at an efficiency of ∼31%. Nonspecific uptake was defined using 10 μM Ro4-1248 and typically ranged from 1–2% of total accumulated counts (typically ∼10% of total counts added). Assays were performed with conditions in duplicate or triplicate for each sample. Data in each assay were normalized to the mean control (maximal) uptake level within the respective assay without background subtraction, and the data from three separate experiments were pooled and analysed using GraphPad Prism (version 5.0, GraphPad Software, La Jolla, CA) with a four parameter logistic model.

### dVMAT-pHluorin characterization

For both immunolabeling and live imaging experiments at the larval neuromuscular junction (segment A4, muscle 13), the *UAS-dVMAT-pHluorin* transgene was expressed in Type Ib nerve terminals using the elav-GAL4 expression driver. For immunolabeling, larval fillets were fixed in 4% paraformaldehyde. Immunolabeling was performed in PBS containing 0.1% Triton X 100 detergent and 5% normal goat serum using either a monoclonal antibody to GFP (1:500, Invitrogen Life Technologies, Carlsbad, CA) or rabbit antiserum to *Drosophila* synaptotagmin 1 (1:500 dilution, gift of N. Reist, Colorado State University) followed by the appropriate secondary antibodies (1:1,000 dilution) conjugated to either Alexa Fluor 488 or Alexa Fluor 555 (both from Jackson ImmunoResearch Laboratories, West Grove, PA), respectively. Images represent projected Z-series acquired via confocal imaging (1 μm/section). For stimulated release experiments, third instar larvae were prepared in chilled calcium-free HL3.1 saline (70 NaCl, 5 mM KCl, 4 mM MgCl_2_, 10 mM NaHCO_3_. 5 mM trehalose, 115 mM sucrose, 5 mM HEPES, adjusted to pH 7.32^72^) and recordings made in HL3.1 supplemented with 2 mM CaCl_2_ and 7 mM L-glutamic acid (with the latter to inhibit muscle contractions). The stimulus (40 Hz, 10 V, 2 s) was applied using a suction electrode in contact with the free nerve root and stimulation-induced changes in dVMAT-pHluorin fluorescence were imaged at a rate of 20 images/s.

To characterize the pH response properties of dVMAT-pHluorin in DA nerve terminals of fly brain, we treated with 40 mM KCl to stimulate exocytic release of the biosensor across a range of extracellular pH's (pH 5.5–8.3) using a modified version of AHL with equimolar substitution of KCl for NaCl. To estimate the intraluminal pH of intact dopaminergic synaptic vesicles in intact brain, we generated a pH calibration curve using dVMAT-pHluorin and a buffered cocktail of ionophores comprised of 20 μM FCCP, 10 μM valinomycin, 10 μM nigericin as well as 1 μM bafilomycin A1 and 0.1% Triton X-100(final concentrations)^73^ across a range of pH's (pH 5.5–8.0) to permeabilize the vesicles and thus equilibrate the intraluminal vesicular pH with the respective extracellular pH. To prevent exocytic release of vesicles in response to ionophore treatment, these experiments were performed in flies co-expressing tetanus toxin light chain in the same dopamine terminals. We generated a pH calibration curve by graphing pH versus Δ*F*/*F*_initial_ where *F*_initial_ was obtained while superfusing AHL (pH 7.5) for 10 min without the ionophore cocktail. Points chosen for the calibration curve were 2 min following complete bath exchange with the respective cocktail and fitted with GraphPad Prism with a logistic model (Hill coefficient=1). For improved stability in these experiments, AHL was modified to include 9 mM HEPES (pH 6.5–8.3) or 9 mM MES (pH 5.0–6.0) and NaHCO_3_ was excluded.

To determine the fraction of dVMAT-pHluorin expressed on the cell surface, we used previously described methods^31^,^33^. Specifically, we subjected whole, *ex vivo*brain preparations expressing dVMAT-pHluorin in presynaptic DA nerve terminals to a brief acid wash (100 s, 25 °C, pH 5.5) to quench fluorescence from cell surface-expressed biosensor, followed by recovery of fluorescence after wash-out (25 °C, pH 7.5). To confirm these values, we used complementary NH_4_Cl alkalization (50 mM, 25 °C, pH 7.5, 60 s) to visualize the intracellular vesicular dVMAT-pHluorin pool combined with KCl treatment (40 mM, 25 °C, pH 7.5, 60 s) to visualize both intracellular and externalized cell surface dVMAT-pHluorin fluorescence. Using the brief acid wash method, we found that the percentage of dVMAT-pHluorin on the cell surface under basal conditions was 14.5%±2.5%, comparable to 17.7%±5.4% determined with the complementary NH_4_Cl alkalization method.

### Imaging

An isolated, *ex vivo* whole adult fly brain preparation was obtained by rapid removal and microdissection of the brain from decapitated flies as previously described^16^. This whole brain preparation was placed in a recording chamber (JG-23, Warner Instruments, Hamden, CT) with continuous flow of AHL. This experimental system affords facile manipulation of drug concentrations. The timing by which drug solutions equilibrated in the imaging chamber was determined by flowing an auto-fluorescent green dye dissolved in PBS buffer (1:100 dilution) under conditions identical to those experimentally used to deliver drugs to fly brains. Brain preparations were imaged on an Ultima multiphoton laser scanning microscope (Prairie Technologies Bruker Corporation, Middleton, WI) using either a 63 × (0.9 numerical aperture (NA)) or a 20 × (1.0 NA) water immersion objective lens (Carl Zeiss Microscopy LLC, Thornwood, NY). The illumination source was a Coherent Chameleon Vision II Ti: Sapphire laser (Coherent, Inc., Santa Clara, CA) and we typically used <5 mW mean power at the sample. Fluorescent emission was collected using a 460 nm/50 nm FWHM bandpass emission filtre for FFN206 (*λ*_ex_=820 nm) and 525/50 nm FWHM bandpass filtre for dVMAT-pHluorin (*λ*_ex_=920 nm). When measuring the effect of FFN206 on dVMAT-pHluorin brightening, there was no cross-talk of the FFN206 signal into the dVMAT-pHluorin 525 nm/50 nm imaging channel when using *λ*_ex_=920 nm. We tested for spectral bleed-through and found that even a 1,000 μM solution of FFN206 generated no detectable fluorescence when using dVMAT-pHluorin excitation parameters (*λ*_ex_=920 nm, ∼1.5 mW mean power at sample). R3846 multi-alkali photomultiplier-tube detectors (Hamamatsu Photonics, Middlesex, NJ) were used for both FFN206 and dVMAT-pHluorin imaging. Data acquisition was performed with Prairie View software (version 4.0.29, Prairie Technologies Bruker Corporation, Middleton, WI).

Fixed larval fillet preparations were imaged on a Zeiss LSM5 Pascal Laser Scanning confocal microscope equipped with a Zeiss 63 × Neofluor, 1.3 NA oil immersion objective lens. Live imaging of these larval fillets was conducted using a Zeiss Axio Examiner Z1 microscope equipped with a cooled back-illuminated electron multiplying CCD camera (Andor iXon3 897, Andor, South Windsor, CT) and DG4 light source (Sutter Instrument, Novato, CA) with a GFP Brightline Filter Set (Semrock, Rochester, NY) and Zeiss Achroplan 100 × (1.0 NA) water-immersion objective lens.

### Image processing and analysis

For experiments involving FFN206 or VMAT-pHluorin imaging in adult fly central brain, maximum-intensity z-projections were generated and quantified using the Fiji/ImageJ image processing package (National Institutes of Health, Bethesda, MD). Unless indicated otherwise, we normalized changes in images' fluorescence intensity by using a ratio of fluorescence intensity change (Δ*F*, calculated as *F*−*F*_baseline_) relative to the maximal fluorescence intensity change (Δ*F*_max_, calculated as *F*−*F*_max_) based on treatment with chloroquine (100 μM, 25 °C), Δ*F*/Δ*F*_max_. In calibrating dVMAT-pHluorin's pH-dependent fluorescence intensity changes in adult central brain, we normalized the respective fluorescence values to a Δ*F*_max_ derived from 40 mM KCl, pH 8.3. dVMAT-pHluorin fluorescence intensity changes in larval fillet preparations during electrical stimulation were expressed as Δ*F*/*F*, which was calculated as (*F*−*F*_baseline_)/*F*_baseline_ where *F*_baseline_ is the average of intensities 2 s before stimulus. Maximal Δ*F*/*F* in this preparation was calculated as (*F*_peak_−*F*_baseline_)/*F*_baseline_, where *F*_peak_ is the average of the 10 frames (0.5 s) acquired immediately following cessation of stimulation.

For curve fittings, images were corrected for background fluorescence and subsequently normalized to initial predrug treatment MB-MV1 fluorescence (*F*_*i*_) using a custom program written in MATLAB (version R2012b, Mathworks, Natick, MA) and described in further detail elsewhere (Aguilar *et al.*, in preparation). The decay time constant (*τ*_decay_) and decay half-time (*t*_1/2_) for FFN206 destaining were estimated using the least-squares method to fit fluorescence values to a single-exponential function preceded by a plateau phase: *F*/*F*_*i*_(*t*)=(*F*_*i*_-Plateau) *e*^(*−*(*t−t*^_*0*_^)/*τ*decay)^+Plateau where *t*_1/2_=*τ*_decay_ ln2 and where F/F_*i*_(*t*) is the normalized fluorescence value as a function of time. Similarly, the time constant for decay of dVMAT-pHluorin fluorescence following electrical stimulation was estimated by fitting Δ*F*/*F* values to a single exponential function (using GraphPad Prism). The time constant (Tau, or 1/k) was calculated using the equation *Y*=(*Y*_0_−*Y*_p_)*e*^*−kt*^ where *Y*_0_ represents the peak after the stimulus and *Y*_p_ represents the asymptotic, plateau value of *Y* at *t*=∞ with *k* as the rate constant of decay. The decay was represented as *t*_1/2_ as calculated from the derived curve.

The signal to noise ratio (SNR) for FFN206 fluorescent signal was calculated as a ratio of the average FFN206 fluorescence in the MB-MV1 region to the standard deviation of background fluorescence. The dVMAT-pHluorin SNR was calculated as the ratio of the CQ-induced peak fluorescence to the s.d. of baseline fluorescence pre-drug treatment in MB-MV1. The dynamic range of dVMAT-pHluorin fluorescence intensity was calculated as a ratio of peak fluorescence intensity to initial fluorescence in response to KCl stimulation. All data were graphed using GraphPad Prism.

### Larval locomotion assay

Two hundred flies (3:2, females:males) of the respective genotype were placed in bottles filled with standard medium and permitted to lay eggs with experiments commencing on the fourth day of egg-laying. twenty to 30 early third instar larvae (82- to 86-h old) per experimental group were washed with distilled water and placed onto 70% yeast paste (vehicle treatment) or 70% yeast paste in 60 mM amphetamine (Sigma, St Louis, MO) for 1 h (18 °C). Food coloring was added to the yeast paste to ascertain whether the larvae fed on the provided paste. Fed larvae were subsequently transferred onto 100-mm Petri dishes filled with 1% agar dissolved in distilled water. Each dish containing a set of 1–3 larvae was placed on a cool-operated, evenly illuminated fluorescent light box positioned underneath a video camera (Dalsa PT-41-04M60, Teledyne, Dalsa, Waterloo, Ontario, Canada), which captured a high-contrast video image of larval profiles over a featureless background. Larvae were acclimated on the agar plate for 1 min followed by 1 min of data acquisition in a designated behaviour room (23–25 °C, 35–40% humidity). We used the Multi-Worm Tracker and Choreography software packages (open source availability) to track and quantify larval movement.

### Rodent behaviour

See Supplementary Methods

### Statistical analyses

Statistical significance for the larval behavioural data were determined by one-way analysis of variance (ANOVA; α=0.05) with a *post hoc* two-sided Dunnett *t*-test for pairwise comparisons; SPSS (version 18.0, IBM, Armonk, NY) was used for all statistical analyses. The coefficient of determination (*R*^2^) corresponded to fitting experimentally-derived FFN206 destaining curves to a one phase exponential decay preceded by a plateau phase; all curve fittings were plotted using GraphPad Prism. For analysis of drug effects on dVMAT-pHluorin fluorescence, respective treatment conditions were compared to vehicle via one-way ANOVA (*α*=0.05) followed by Bonferroni *post hoc* tests to compare between-group differences. For analysis of the rodent behavioural data, statistical significance was assessed by one- or two-way repeated-measures ANOVA (α=0.05) followed by *post hoc* Bonferroni *t*-tests for all pairwise comparisons. IC_50_ values were computed using a nonlinear, least-squares regression analysis using GraphPad Prism. Affinities (*K*_i_ values) were calculated using the Cheng–Prusoff equation^74^.

## Additional information

**How to cite this article:** Freyberg, Z. *et al*. Mechanisms of amphetamine action illuminated through optical monitoring of dopamine synaptic vesicles in *Drosophila* brain. *Nat. Commun.* 7:10652 doi: 10.1038/ncomms10652 (2016).

## Supplementary Material

Supplementary Information Supplementary Figures 1-13, Supplementary Table 1, Supplementary Note 1, Supplementary Discussion and Supplementary References.
