# Supplementary material for: Mechanisms of amphetamine action illuminated through optical monitoring of dopamine synaptic vesicles in Drosophila brain
Source: Nat Commun. 2016 Feb 16;7:10652. doi: 10.1038/ncomms10652 (PMC4757768; doi:10.1038/ncomms10652)
Supplement: Supplementary Information — Supplementary Figures 1-13, Supplementary Table 1, Supplementary Note 1, Supplementary Discussion and Supplementary References. [file ncomms10652-s1.pdf]

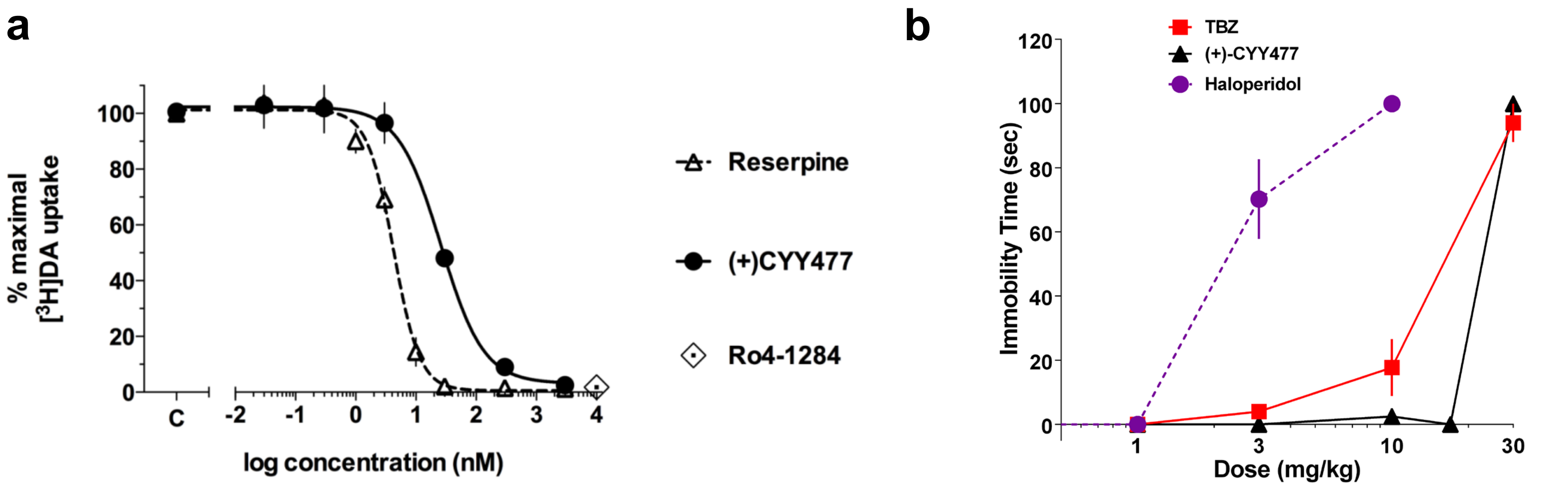

Supplementary Figure 1. Characterization of (+)-CYY477 *in vitro* and *in vivo*. (a)(+)-CYY477 and reserpine inhibition of [^{3}H]dopamine uptake by rat VMAT2 in HEK cells. 293-rV2 cells, HEK293 cells stably transfected with rat VMAT2, were incubated with [^{3}H]dopamine in the presence of 10 µM digitonin and 5 mM ATP (see Methods). Co-incubation with VMAT inhibitors reserpine and (+)-CYY477 dose-dependently blocked [^{3}H]dopamine uptake with inhibitory potencies of 4.2 nM (95% CI: 3.7-4.9 nM) and 26.1 nM (95% CI: 17.2-39.4 nM), respectively. These inhibitors reached the same levels of inhibition as 10 µM Ro4-1284, which we used to define non-specific uptake. Error bars represent SEM with 6-10 replicates for each inhibitor concentration pooled from 3 separate assays; C (control) represents absence of inhibitor within the assay. **(b)** Cataleptic effects of (+)-CYY477, its parent compound tetrabenazine (TBZ), and haloperidol in Swiss-Webster mice. (+)-CYY477 produced catalepsy at 30 mg/kg comparable to TBZ; the control haloperidol produced catalepsy at much lower doses. Ordinates: Time until subject removed its forepaws from the bar. Maximal time allowed was 100 sec. Abscissae: Dose in mg/kg, log scale. Each point represents the mean ± SEM (N=4). All injections were i.p. with measurements made 30 min after injection.

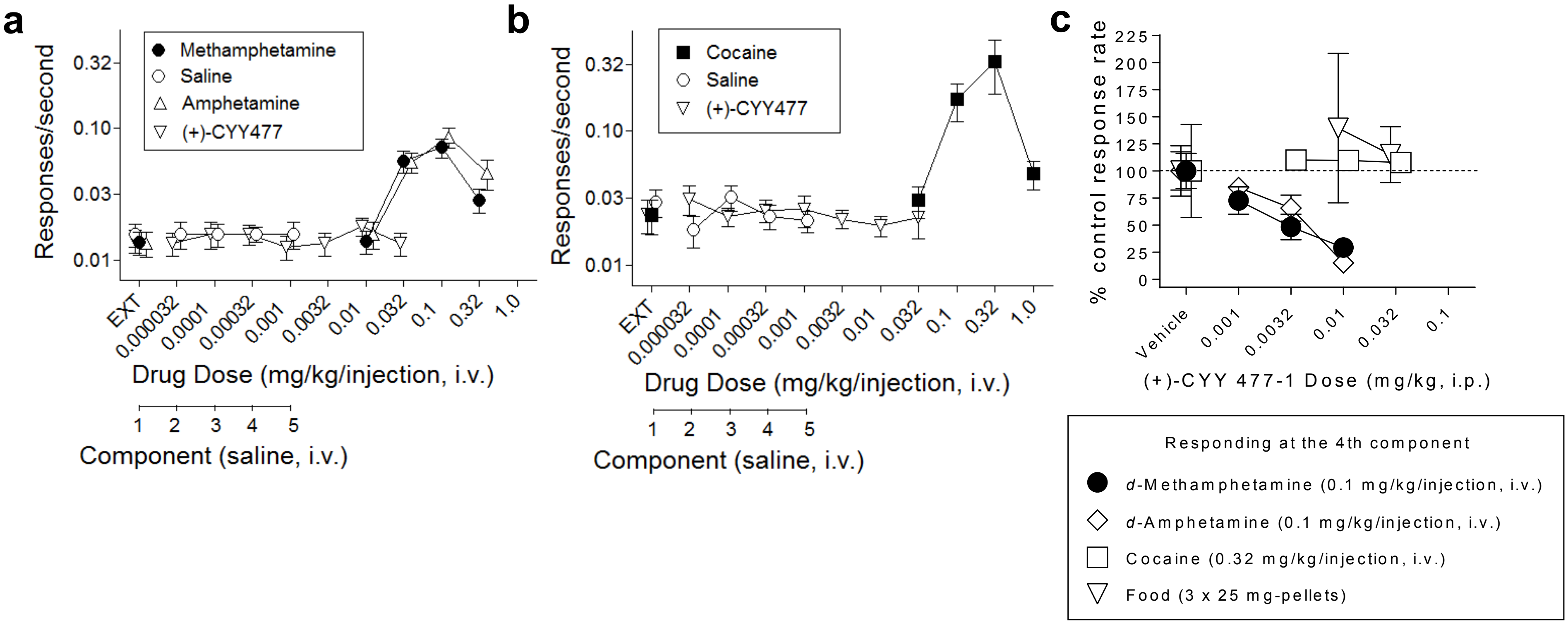

Supplementary Figure 2. (+)-CYY477 substitution alone does not stimulate rodent drug self-administration above vehicle levels. (a) Application of increasing (+)-CYY477 concentrations in rats trained to self-administer methamphetamine did not increase self-administration response rates compared to the saline control ($p \geq .139$). By contrast, methamphetamine injections caused dose-dependent patterns of drug self-administration: 0.032 mg/kg/injection ($t=6.85$; $p<.001$), 0.1 mg/kg/injection ($t=9.42$; $p<.001$) and 0.32 mg/kg/injection ($t=2.18$; $p=.041$). Similarly, amphetamine injections caused response rates greater than saline: 0.032 mg/kg/injection ($t=4.98$; $p<.001$), 0.1 mg/kg/injection ($t=8.80$; $p<.001$) and 0.32 mg/kg/injection ($t=3.71$; $p=.002$). Two-way repeated measures ANOVA indicated a significant effect of drug (vs. saline), component and interaction of the two on response rate for methamphetamine relative to saline [$F(1,20)=34.1$, $p=.002$; $F(4,20)=30.6$, $p<.001$; $F(4,20)=24.6$, $p<.001$]. Similarly, when amphetamine was substituted for methamphetamine, two-way repeated measures ANOVA indicated a significant effect of drug (vs. saline), component and interaction of the two on the response rate for amphetamine relative to saline [$F(1,20)=27.3$, $p=.003$; $F(4,20)=19.7$, $p<.001$; $F(4,20)=20.1$, $p<.001$]. **(b)** In rats trained to self-administer cocaine, no (+)-CYY477 doses caused drug self-administration rates greater than vehicle (saline) injections. ($p \geq .115$) **(c)** (+)-CYY477 was more potent in decreasing self-administration response rates for methamphetamine and amphetamine than cocaine injections or food presentation. One-way ANOVA indicated a significant difference between groups [$F(3,20)=5.63$, $p=.006$]. Post-hoc analysis with Bonferroni t-tests indicated that control response rates maintained by food presentation were significantly higher than those methamphetamine and amphetamine injections ($t \geq 3.43$, $p \leq .016$). For all panels, a specified dose of 0 mg/kg of each test compound indicated saline vehicle pretreatments. Abscissae: mg/kg/injection of drug dose and component (**Panels a & b**) and mg/kg of (+)-CYY477 administered i.p. (**Panel c**), log scale. Ordinates: Response rates as actual values (responses/s, **Panels a & b**) and % control response rates (sessions prior to drug tests, **Panel c**). Each point represents the mean ± SEM of response rates on the active lever in 6 rats; EXT: extinction (no injection).

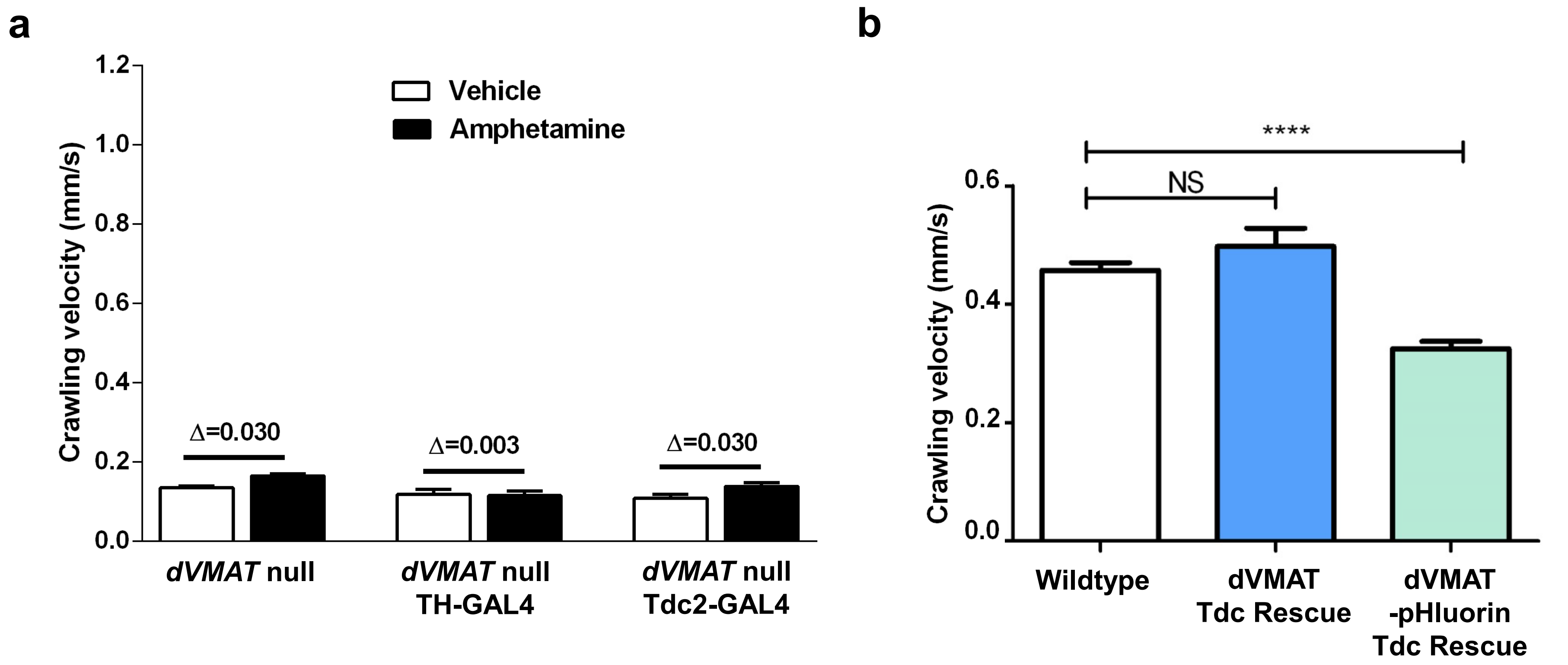

Supplementary Figure 3. Effects of expression drivers and dVMAT-pHluorin on larval locomotion in *dVMAT* null mutants. We compared larval crawling velocity in *dVMAT* null mutants to strains expressing either TH-GAL4 or Tdc2-GAL4 expression drivers (without the accompanying *UAS-dVMAT* transgene) in the *dVMAT* null background. Basal locomotion in all three strains fed vehicle (yeast alone) were low compared to wildtype (see **Fig. 2a**). *dVMAT* null larvae expressing TH-GAL4 alone exhibited no significant amphetamine-stimulated hyperlocomotion [$p>.10$; N=22 (vehicle), N=19 (amphetamine); independent sample t-test]. Tdc2-GAL4 expression resulted in a small increase in locomotion in response to amphetamine [$t(53)=2.2$, $p=.032$; N=26 (vehicle), N=29 (amphetamine); independent sample t-test]. However, this small amphetamine response matched that in the dVMAT null strain ($\Delta=0.03$ mm/s for both groups), suggesting that the Tdc2-GAL4 did not have a direct effect on the response. Differences in average larval crawling speed between vehicle and amphetamine-fed groups are indicated above each respective genotype. **(b)** To verify that dVMAT-pHluorin functions as a monoamine transporter, we compared basal crawling velocity in wildtype versus *dVMAT* null mutants where either dVMAT or dVMAT-pHuorin expression was selectively rescued using the Tdc2-GAL4 expression driver. Rescue with the *UAS-dVMAT* transgene restored basal locomotion to levels not significantly different from wildtype [$p>.05$; N=36 (wildtype), N=20 (dVMAT Tdc Rescue); one-way ANOVA]. Rescue with the *UAS-dVMAT-pHluorin* transgene resulted in less efficient rescue of basal locomotion with a 29% reduction in basal locomotion compared to wildtype larvae [$F(2, 91)=29.66$, $p<.01$; N=38 (dVMAT-pHluorin Tdc Rescue); one-way ANOVA]. Every condition represents the mean ± SEM with all experiments conducted on ≥2 separate occasions.

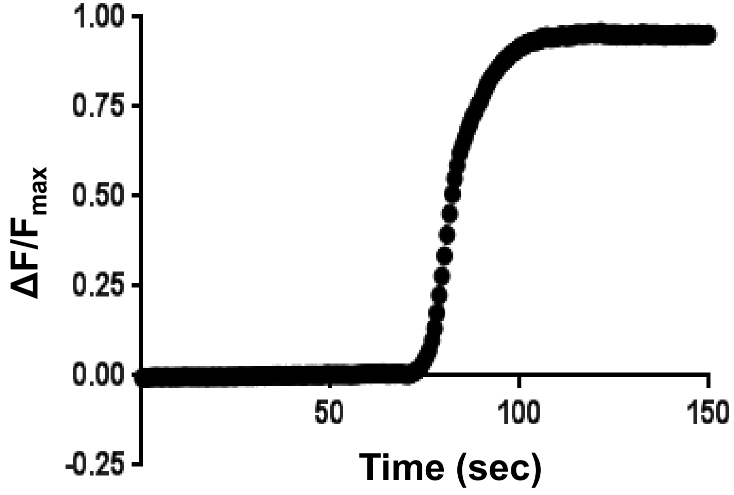

Supplementary Figure 4. Optical determination of drug delivery kinetics to a whole, *ex vivo* fly brain preparation. To characterize the drug delivery flow rate in our experimental system, we diluted a fluorescent green dye in PBS buffer and determined the rate at which the solution equilibrated using identical experimental parameters to those employed to deliver drugs to fly brains (N=7 separate experiments). Quantitative imaging revealed that dye was first detected in the flow chamber 75.41±1.14 s after start of administration. λ_{ex}=820 nm, λ_{em}=460/50 nm FWHM.

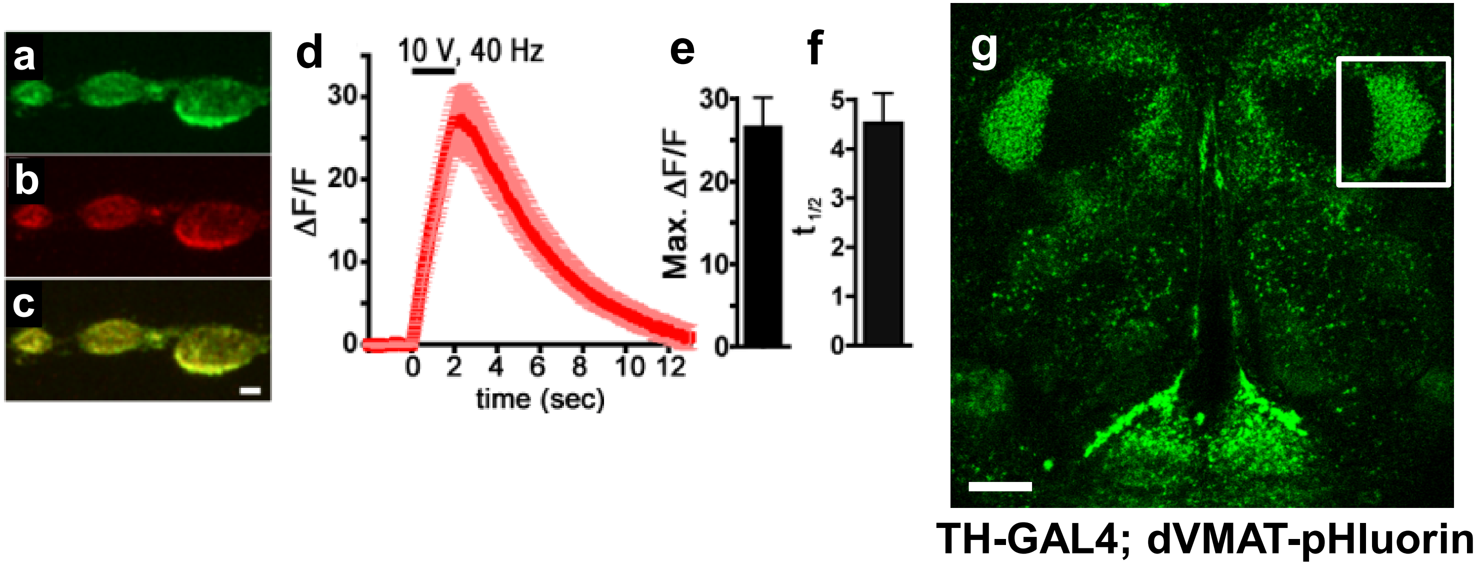

Supplementary Figure 5. dVMAT-pHluorin localizes to vesicles. (a-c) Immunostaining of dVMAT-pHluorin and synaptotagmin 1, a secretory vesicle marker. dVMAT-pHluorin (in green, **Panel a**) and synaptotagmin 1(in red, **Panel b**) co-localized closely (**Panel c**, merged image with overlap in yellow) in Type Ib terminals of the larval neuromuscular junction. Images are from projected Z-series acquired in 1 μm steps using confocal imaging; scale bar = 2 μm. (**d-f**) Monitoring exocytic release with dVMAT-pHluorin fluorescence. (**d**) Electrical stimulation (40 Hz, 10 V, 2 sec, indicated with a black bar in **Panel d**) of Type Ib nerve terminals expressing dVMAT-pHluorin resulted in a rapid increase in fluorescence (maximum ΔF/F shown in **Panel e**) followed by exponential decay (quantified as $t_{1/2}$ in **Panel f**) was consistent with the expected exocytic cycle of secretory vesicles. (**g**) dVMAT-pHluorin in presynaptic DA nerve terminals closely resembled FFN206 labeling in TH rescue brain (compare white box for MB-MV1 region in **Fig. 3d**). **Panels d-f:** data represented as mean ± SEM, N=12 samples; λ_{ex} = 430-472 nm, λ_{em} = 520-535 nm. Scale bar = 25 μm. **Panel g:** Image is from a projected Z series of coronal sections acquired with similar settings and of comparable depth; λ_{ex} = 920nm, λ_{em} = 525/50 nm FWHM. Scale bar = 25 μm. Results were from N≥3 independent experiments.

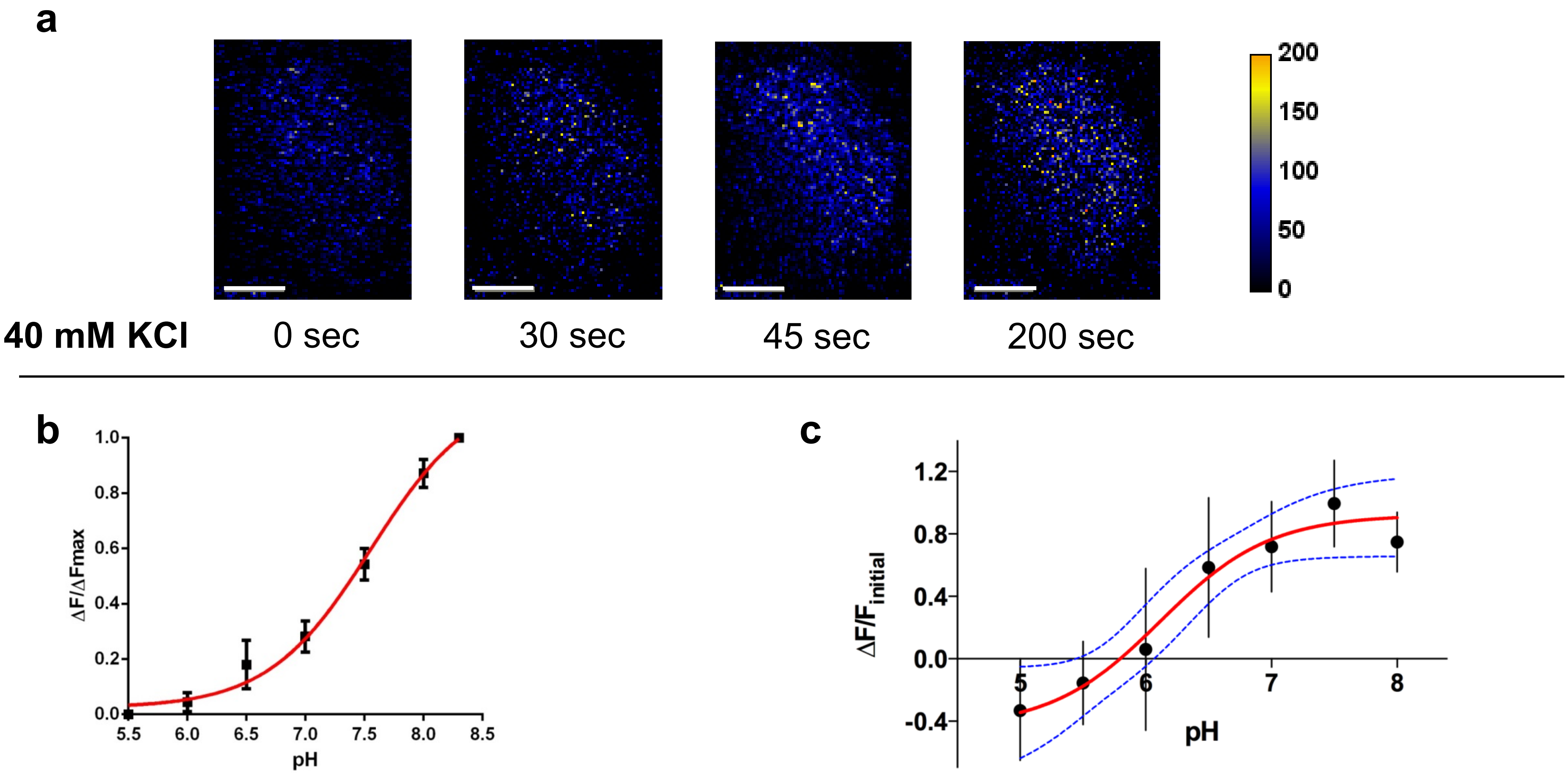

Supplementary Figure 6. dVMAT-pHluorin characterization. (a) Multiphoton imaging of dVMAT-pHluorin expressed in presynaptic DA nerve terminals of a whole, *ex vivo* fly brain preparation treated with 40 mM KCl to induce synaptic vesicle exocytosis. Quantitative imaging of the MB-MV1 region revealed progressive brightening of the dVMAT-pHluorin signal over time. This example is representative of 3 experiments. Images are from projected Z-series acquired in 1 μm steps; scale bar 10 μm, arbitrary fluorescence units. **(b)** dVMAT-pHluorin fluorescence intensities in presynaptic DA nerve terminals of MB-MV1 were measured following exocytosis due to treatment with 40 mM KCl buffers of differing pH. The pH calibration curve is best fit to a logistic dose response curve (in red, R^2=0.96) revealing a pKa of 7.5 ± 0.2. Data were normalized to pH 8.4 in each of 4 separate experiments and pooled. **(c)** Changes in dVMAT-pHluorin fluorescence relative to the initial intensity at pH 7.5 were used to estimate vesicle intraluminal pH. We calibrated intensities to ionophore-containing buffers of known pH in *ex vivo* brain preparations from flies selectively expressing tetanus toxin light chain in DA nerve terminals to prevent vesicular exocytosis during treatment. The X-intercept of the pH calibration curve revealed the resting pH of the vesicles to be pH 5.8 (95% CI: pH 5.45-6.08). The curve (in red) was best fit with a logistic sigmoidal curve (Hill slope = 1, EC_{50} = 6.1; blue lines indicate 95% confidence intervals for the curve fit). Data from 26 brains were used in calculating the curve with N≥3 independent experiments for each pH. For **Panels b & c:** Points represent means ± SEM. λ_{ex} = 920 nm, λ_{em}= 525/50 nm FWHM.

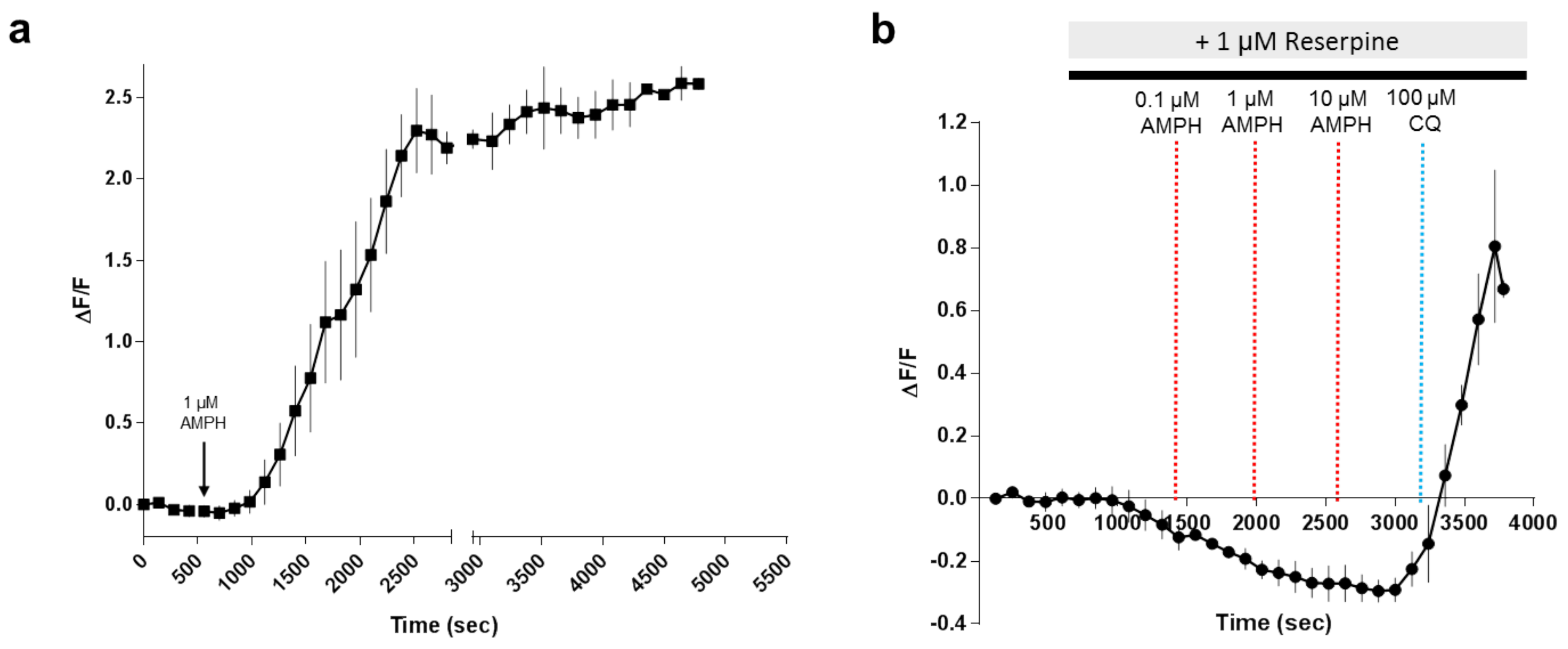

Supplementary Figure 7. Time courses of bath application of drugs. (a) Bath application of amphetamine causes a sustained increase in intraluminal DA vesicle pH. Amphetamine (1 μM) treatment of *ex vivo* whole brain preparations selectively expressing dVMAT-pHluorin in presynaptic DA nerve terminals by bath application produced a progressive increase in fluorescence intensity of the probe followed by a sustained plateau. Arrow indicates onset of drug application. Results are pooled from N=6 separate experiments for time points up to 2800 seconds of treatment and N=2 separate experiments for time points ≥2940 seconds. **(b)** Time course of amphetamine treatment in the presence of VMAT blockade. Despite sequential addition of escalating amphetamine concentrations (0.1-10 μM), ongoing VMAT inhibition by 1 μM reserpine blocked amphetamine-induced vesicle deacidification. Unlike amphetamine, 100 μM chloroquine (CQ) caused a rise in intraluminal pH despite continued VMAT inhibition by reserpine. Dotted lines indicate onset of bath application of the respective drugs. Results are pooled from N=3 separate experiments. In **Panels a & b**, all drugs were applied by bath superfusion and dVMAT-pHluorin fluorescence changes were normalized to the initial fluorescence intensity. Points represent means ± SEM.

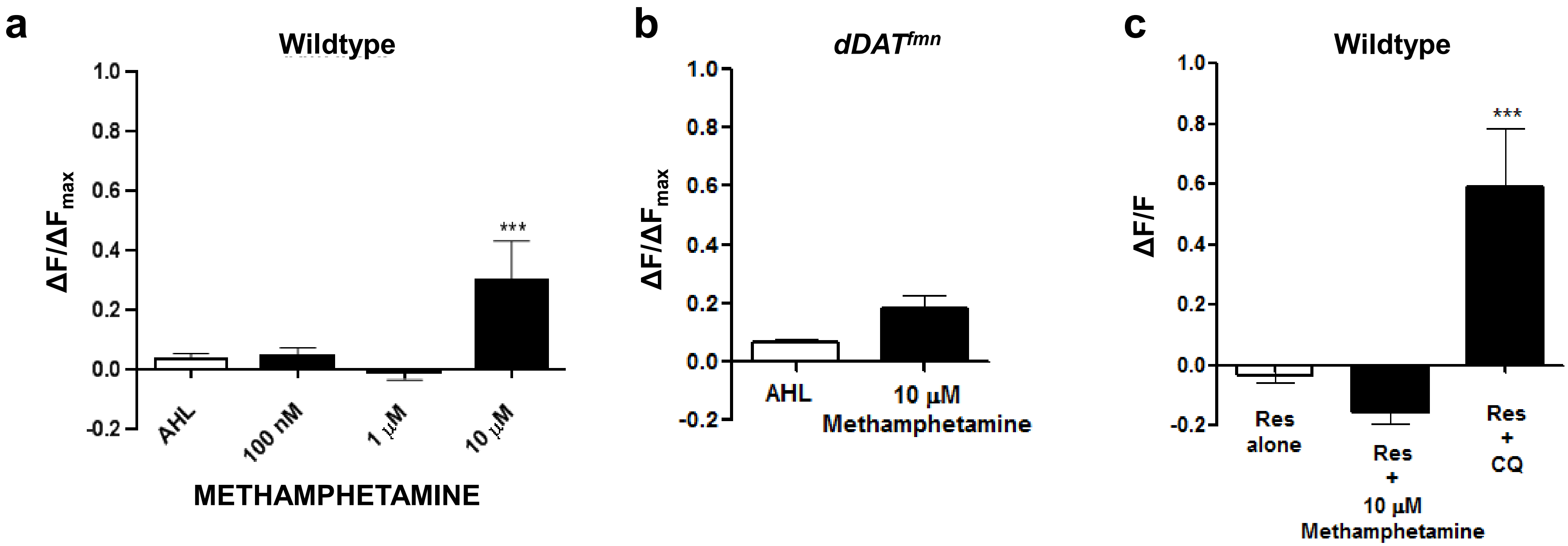

Supplementary Figure 8. Methamphetamine requires functional DAT and VMAT to alkalize vesicles in DA nerve terminals. (a) Methamphetamine treatment (10 μM) caused significant vesicular alkalization in presynpatic DA terminals expressing dVMAT-pHluorin [$p=.002$; $F(3,20)=9.03$, $p=.001$; one-way ANOVA with Bonferroni post-hoc tests]. **(b)** In the *dDAT* null *fumin* genetic background, relevant methamphetamine concentrations (10 μM) did not significantly alkalize vesicles indicated by lack of dVMAT-pHluorin brightening ($p>.05$). **(c)** dVMAT blockade by reserpine (1 μM, 20 min, 25°C) prevented vesicle alkalization by methamphetamine(10 μM) ($p>.05$) but did not block chloroquine (CQ)-induced alkalization (100 μM) [$F(2,21)=12.89$, $p<.001$; 1 μM Reserpine + 100 μM CQ, $p=.001$; one-way ANOVA with Bonferroni post-hoc tests]. Results were obtained from N≥4 separate experiments. All error bars represent SEM.

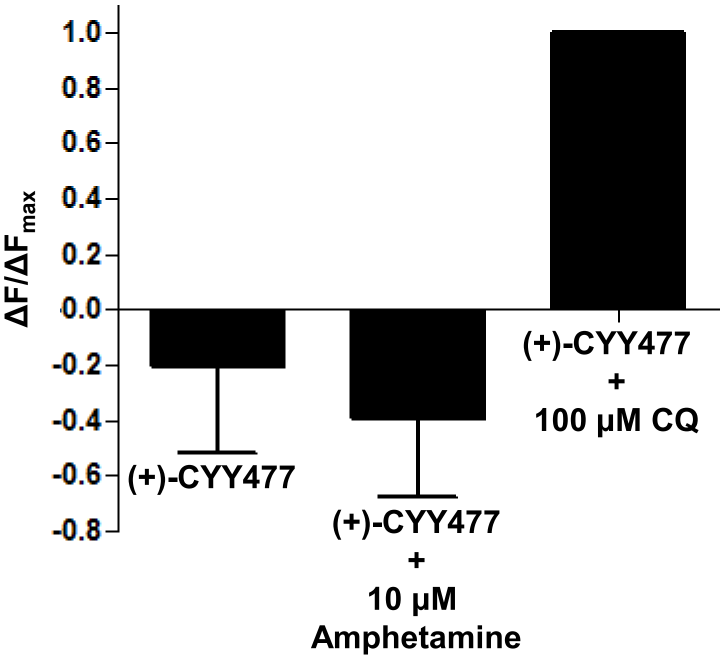

Supplementary Figure 9. (+)-CYY477 blocks amphetamine-induced dopamine vesicle alkalization. dVMAT blockade by (+)-CYY477 (1 µM, 10 min pretreatment, 25°C) prevented vesicle alkalization by treatment with 10 µM amphetamine in the same brains as measured by dVMAT-pHluorin brightening [$F(2,9)=9.74$, $p=.006$; one-way ANOVA with Bonferroni post-hoc analysis] but did not block subsequent alkalization by 100 µM CQ ($p=.008$ difference). Treatment with (+)-CYY477 also caused increased acidification as indicated by an overall decrease in dVMAT-pHluorin fluorescence ($p=.019$). Increases in fluorescence in MB-MV1 regions were normalized to final CQ (100 µM) changes (ΔF_{max}). Data is represented as mean intensities ± SEM in the MB-MV1 region from 4 separate experiments in all 3 conditions.

Column: Phenomenex Gemini C18 5μ 110A 250 x 4.6 mm (P09)
Column temp.: 30
Mobile phase: 60% MeCN/ NH4OAc with CH3COOH (pH=4.5)
Flow rate: 1.0 mL/ min
Injection volumn: 10

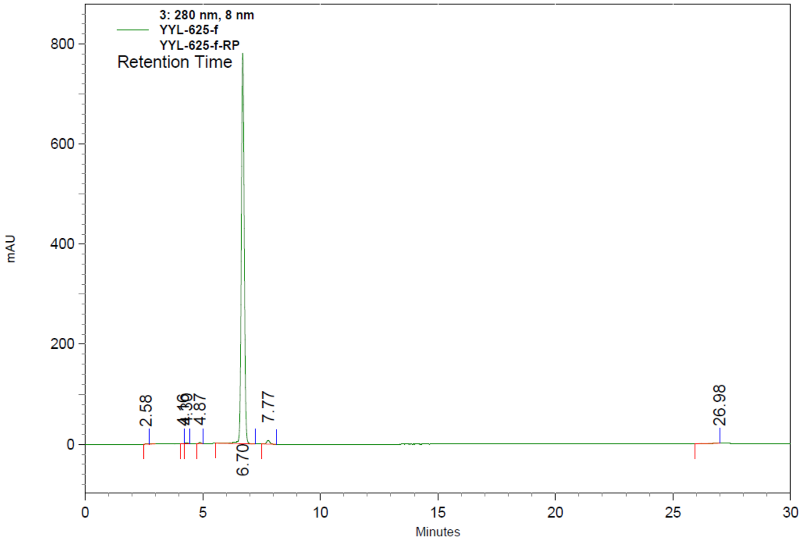

3: 280 nm, 8 nm

Pk #	Retention Time	Area	Area Percent
1	2.58	4125	0.06
2	4.16	3804	0.06
3	4.30	13866	0.20
4	4.87	13708	0.20
5	6.70	6657285	98.36
6	7.77	69493	1.03
7	26.98	5810	0.09
Totals		6768091	100.00

Supplementary Figure 10. RP-HPLC chromatogram of (+)-CYY477. The chemical purity of (+)-CYY477 was determined by high-performance liquid chromatography (HPLC) using Phenomenex Gemini C18 column (5μ, 110Å, 250 × 4.6 mm) at 30°C and 60% acetonitrile in ammonium acetate aqueous solution (pH = 4.5) as the mobile phase.

Column: ChiralPak AD-H 4.6 x 250 mm (DC01)
Instrument: SHIMADZU LC-6AD, SPD-20A
Mobile phase: 20 %IPA/ *n*-Hexane (with 0.2% DEA)
Pressure: 51 kgf/ cm2
Flow rate: 1.0 mL/min
Injection volume (μL): 10
Column temp.: RT (22)

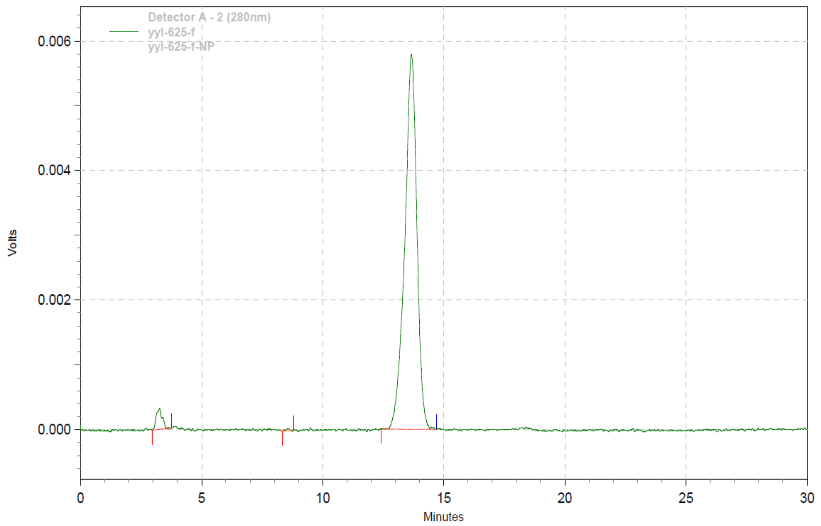

Detector A - 2 (280nm)

Pk #	Retention Time	Area	Area Percent
1	3.25	5604	2.70
2	8.56	345	0.17
3	13.66	201831	97.14
Totals		207780	100.00

Supplementary Figure 11. Chiral HPLC chromatogram of (+)-CYY477. The enantiomeric excess (ee) of (+)-CYY477 was determined by chiral HPLC using a ChiralPak AD-H column (250 × 4.6 mm) at room temperature and 20% 2-propanol in n-hexane (with 0.2% diethylamine) as the mobile phase.

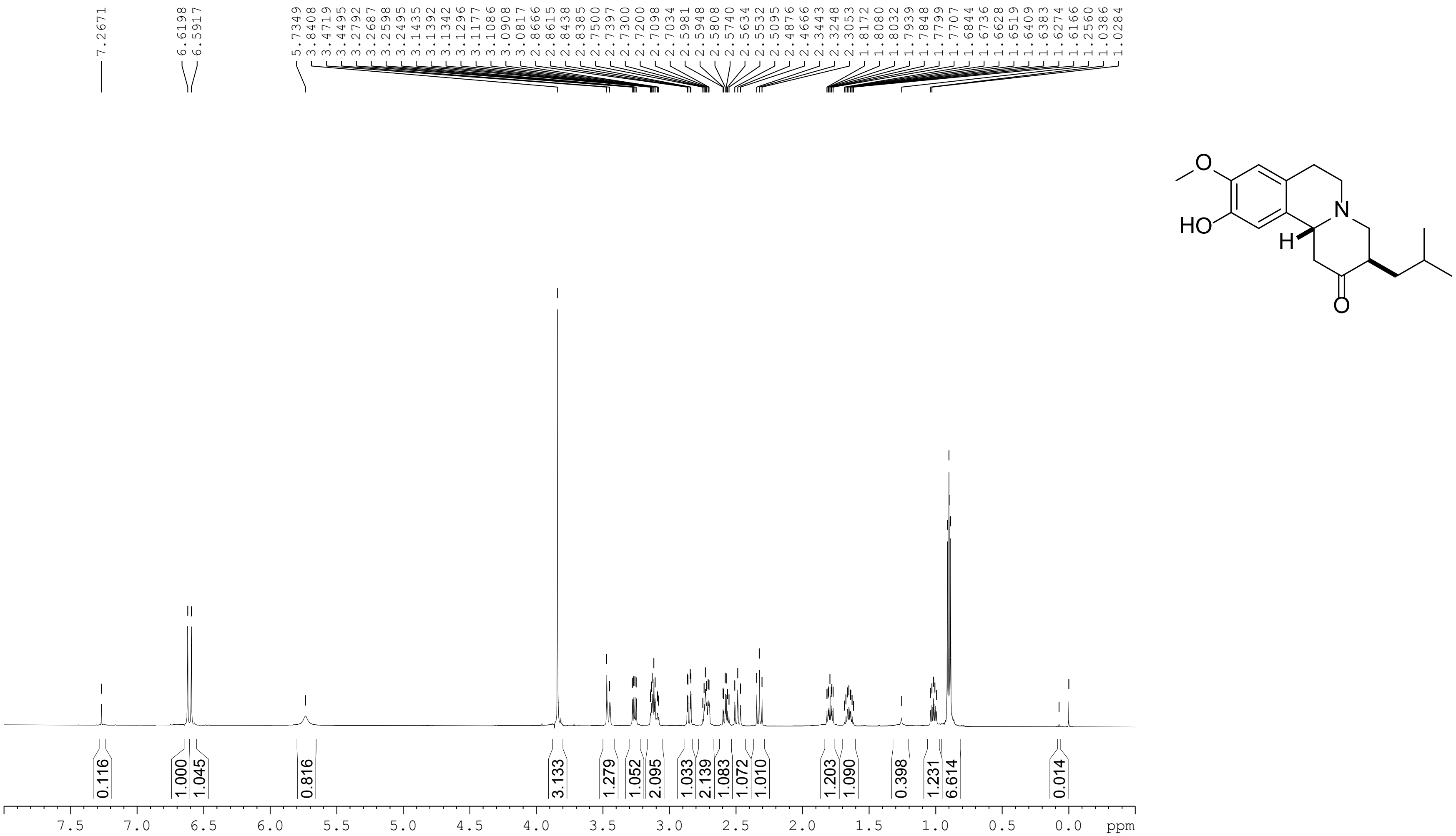

Supplementary Figure 12. ¹H-NMR spectrum of (+)-CYY477. The ¹H-NMR spectra of (+)-CYY477 [600 MHz, $CDCl_3$; 20 mg (+)-CYY477 in 0.7 mL $CDCl_3$ containing 0.03% tetramethylsilane (TMS)] were recorded on a Bruker AVIII-600 FT-NMR spectrometer. Chemical shifts were expressed in parts per million (ppm) on the δ scale relative to the TMS internal standard.

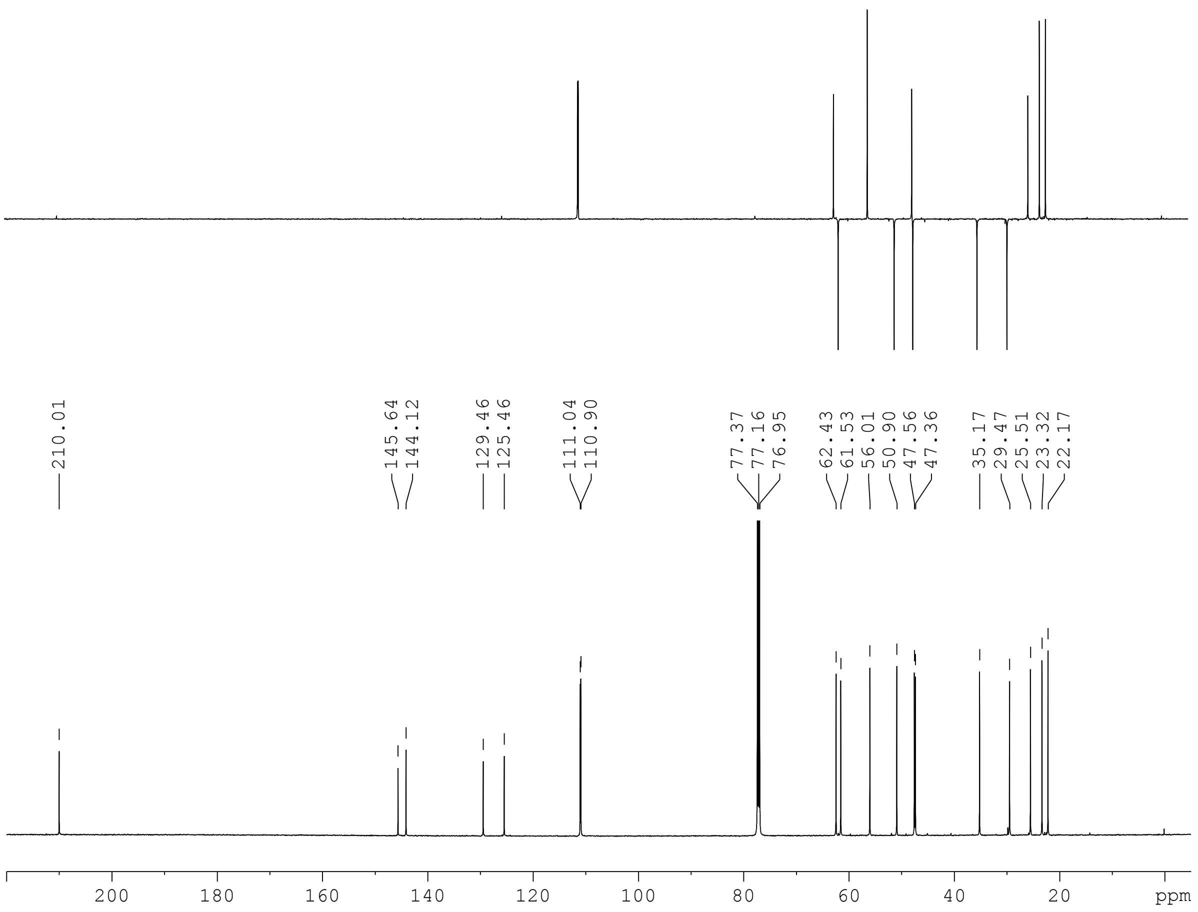

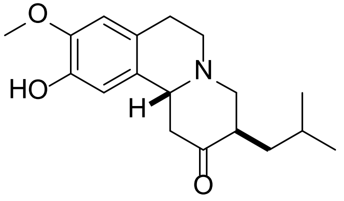

Supplementary Figure 13. ^{13}C-NMR spectra of (+)-CYY477. The ^{13}C-NMR spectra of (+)-CYY477 [150 MHz, $CDCl_3$; 20 mg (+)-CYY477 in 0.7 mL $CDCl_3$ containing 0.03% TMS] were recorded on a Bruker AVIII-600 FT-NMR spectrometer. Chemical shifts were expressed in parts per million (ppm) on the δ scale relative to the TMS internal standard.

Supplementary Table 1. (+)-CYY477 transporter binding screen. (+)-CYY477 competition with the binding of radioligands labeling VMAT2, DAT, SERT, and NET. Value for VMAT2 is K_i value (±SEM) for displacement of the radioligand as determined with four independent replications. The K_i value at the other respective transporters was determined to be greater than 100 μM in two replications.

**Compound**	**VMAT2 K_i Value (nM)** **[^{3}H]Dihydrotetrabenazine**	**DAT K_i Value (nM)** **[^{3}H]WIN 35,428**	**SERT K_i Value (nM)** **[^{3}H]Citalopram**	**NET K_i Value (nM)** **[^{3}H]Nisoxetine**
(+)-CYY477	7.18 ± 1.14	>100 μM	>100 μM	>100 μM

Supplementary Note 1: Chemical Synthesis and Characterization

Experimental procedures for the Synthesis of (+)-CYY477

Design and screening of (+)-CYY477

Tetrabenazine (TBZ) has been used as an antidyskinetic and antipsychotic medication for decades[1]. Indeed, Xenazine® [(±)-TBZ] was approved by the U.S. Food and Drug Administration for treatment of chorea associated with Huntington's disease in 2008. Although this TBZ formulation is composed of both enantiomers, only the dextro-isomer of TBZ is a potent VMAT2 ligand, whereas the levo-isomer is less active at VMAT2[2]. Therefore, in an effort to discover novel potent and selective ligands for VMAT2, a series of enantiomeric 10-substituted-TBZ derivatives was designed and synthesized (described below). We screened these new compounds in VMAT2 radioligand binding assays as described previously[3] (see Methods). Briefly, rat brain striatum was homogenized in 20 volumes (w/v) of ice cold buffer using a Brinkman Polytron (setting 6, 20 s) and centrifuged at 30,000 x *g* (10 min, 4°C) to yield membranes. This assay was conducted in 0.5 mL HEPES-sucrose buffer for 60 min at room temperature with each tube containing 2 nM [^{3}H]dihydrotetrabenazine (American Radiolabeled Chemicals, St Louis, MO) and 1 mg striatal tissue (o.w.w.). Nonspecific binding was determined using 20 μM (±)-TBZ. We also used [^{3}H]WIN 35,428, [^{3}H]citalopram or [^{3}H]nisoxetine in similar binding assays to determine whether (+)-CYY477 also demonstrated significant binding affinities at DAT, SERT and NET, respectively. From this screen, we selected (+)-CYY477 based both on its potent VMAT2 binding affinity (K_i = 7.18±1.14 nM) and its high degree of VMAT2 specificity relative to the plasma membrane transporters (K_i > 100 μM at DAT, SERT and NET).

Preparation of (+)-CYY477

General Procedures

NMR spectra were recorded on a Bruker AVIII-600 FT-NMR spectrometer (Bruker Corporation, Fremont, CA). Chemical shifts were expressed in parts per million (ppm) on the δ scale relative to a tetramethylsilane (TMS) internal standard. Electrospray ionization (ESI) mass spectra and HRMS were obtained using Bruker Esquire 2000 and Bruker Daltonik micrOTOF mass spectrometers, respectively. Elemental analyses were performed with a Heraeus varioIII-NCSH instrument (Heraeus Holding GmbH, Hanau, Germany) and were within ±0.4% for the elements indicated. Optical rotations were obtained using a JASCO DIP-370 polarimeter (JASCO Analytical Instruments, Easton, MD) and were reported at the sodium D-line (589 nm) unless otherwise noted. Thin-layer chromatography (TLC) was performed on Merck (art. 5554) silica gel plates (Merck KGaA, Darmstadt, Germany) and visualized under UV light (254 nm) upon treatment with iodine vapor or upon heating after treatment with 5% phosphomolybdic acid in ethanol. Flash column chromatography was performed with Merck (art. 9385) 40–63 μm silical gel 60. No attempt was made to optimize yields.

Synthetic Scheme

Scheme 1

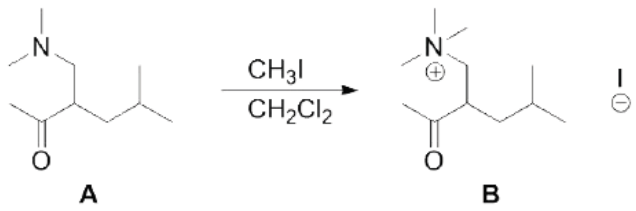

Scheme 2

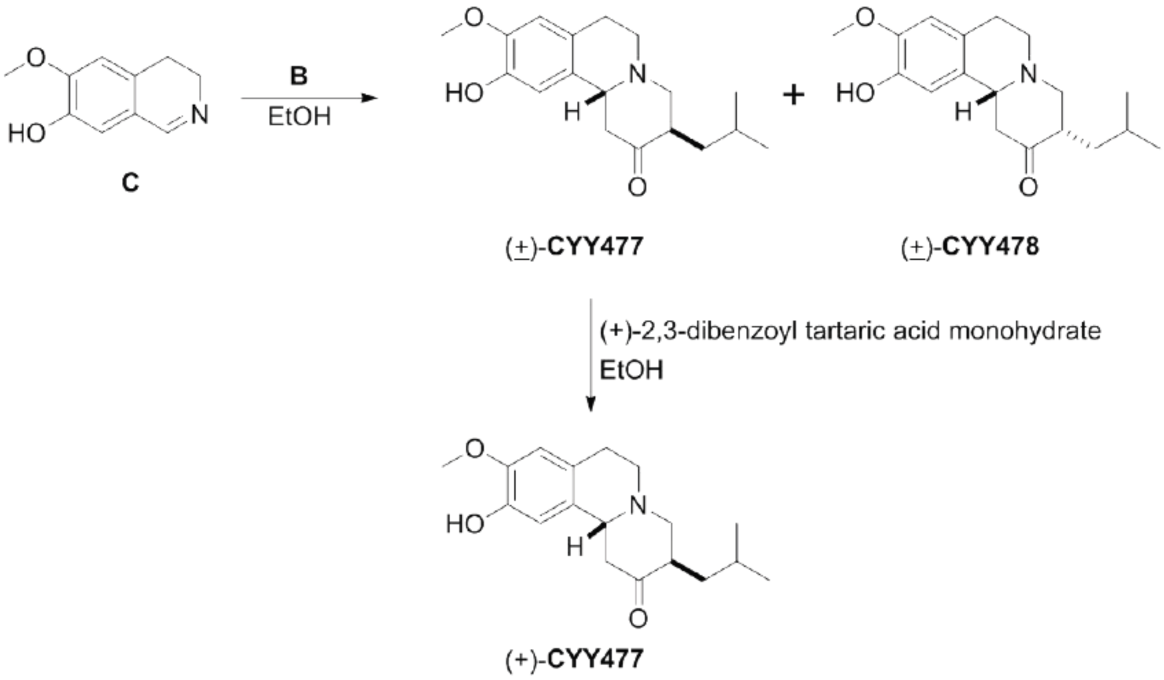

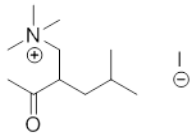

Chemical Formula: $C_{11}H_{24}INO$

Exact Mass: 313.0903

Molecular Weight: 313.2188

2-Acetyl-*N*,*N*,*N*,4-tetramethylpentan-1-aminium iodide (B)[4]

To a solution of 3-((dimethylamino)methyl)-5-methylhexan-2-one[4] (**A**, 2.688 g, 15.69 mmol) in CH_2Cl_2 (25 mL), iodomethane (3.292 g, 23.19 mmol) was added . The mixture was stirred at room temperature under N_2 for 4 h. The resulting white suspension was evaporated to afford a white solid. The crude product was dried under vacuum at 45°C for 2 h to afford compound **B** (4.916 g, 96%). The ^{1}H NMR spectrum was consistent with 2-acetyl-*N*,*N*,*N*,4-tetramethylpentan-1-aminium iodide (**B**) in WO2011/153157A2 (Step 8). ^{13}C NMR (50 MHz, DMSO-d_6) δ 21.5, 22.9, 25.0, 28.6, 39.1, 43.9, 52.4 (3C), 64.1, 208.5; ESIMS calcd for $C_{11}H_{24}NO$ $[M-I]^+$, 186; found, 186.

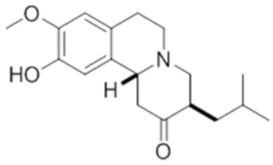

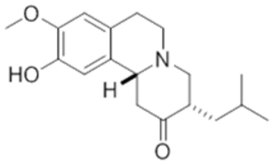

Chemical Formula: $C_{18}H_{25}NO_3$
Exact Mass: 303.1834
Molecular Weight: 303.3960

trans-10-desmethylTBZ *cis*-10-desmethylTBZ

trans-10-Desmethyltetrabenazine [(±)-CYY477] and _cis_-10-desmethyltetrabenazine [(±)-CYY478]

To a suspension of 7-hydroxy-6-methoxy-3,4-dihydroisoquinoline (compound **C**, 2224 mg, 10.55 mmol, purchased from Acros Organics, Product code 277820010) in absolute ethanol (14 mL), compound **B** (4.665 g, 14.69 mmol) was added. The resulting mixture was refluxed under N_2 for 16 h to give an orange suspension. The suspension was cooled to room temperature, and then evaporated to afford an orange solid. The solid was treated with a solution of $NaHCO_{3(sat.)}$ and H_2O (1/1, 100 mL) followed by extraction with CH_2Cl_2 (100 mL × 3). The organic layers were washed with brine, dried over $MgSO_4$, filtered, and evaporated to afford an orange solid. The crude product was purified by flash column chromatography (silica gel, ethyl acetate/*n*-hexane = 1/4~1/1) to afford (±)-**CYY477** (2232 mg, 7.36 mmol, 59%) as a white solid and (±)-**CYY478** (418 mg, 1.38 mmol, 11%) as an ivory paste.

(±)-**CYY477**: R_f = 0.35 (ethyl acetate: *n*-hexane = 1:1); ^{1}H NMR (600 MHz, $CDCl_3$) δ 0.89 (d, *J* = 7.0 Hz, 3H, H-3’), 0.91 (d, *J* = 7.0Hz, 3H, H-3’), 0.99-1.04 (m, 1H, H-1’), 1.62-1.69 (m, 1H, H-2’), 1.77-1.82 (m, 1H, H-1’), 2.32 (t, *J* = 11.7 Hz, 1H, H-4_{ax}), 2.47-2.51 (m, 1H, H-1), 2.55-2.60 (m,1H, H-6), 2.70-2.75 (m, 2H, H-7 and

H-3), 2.86 (dd, *J* = 16.5, 2.9 Hz, 1H, H-1), 3.08-3.13 (m, 2H, H-6, H-7), 3.27 (dd, *J* = 11.5, 6.2 Hz, 1H, H-4_{eq}), 3.46 (bd, *J* = 11.2 Hz, 1H, H-11b), 3.85 (s, 3H, 9-OMe), 5.70 (bs, 1H, 10-OH), 6.59 (s, 1H, H-8), 6.62 (s, 1H, H-11); ^{13}C NMR (150 MHz, $CDCl_3$) δ 22.2 (C-3'), 23.3 (C-3'), 25.5 (C-2'), 29.5 (C-7), 35.2 (C-1'), 47.4 (C-1) , 47.6 (C-3), 50.9 (C-6), 56.0 (-OMe), 61.6 (C-4), 62.4 (C-11b), 110.9 (C-8), 111.0 (C-11), 125.5 (C-11a), 129.5 (C-7a), 144.1 (C-10), 145.6 (C-9), 210.0 (C-2); HRESIMS calcd for $C_{18}H_{26}NO_3$ $[M+H]^+$, 304.1913; found, 304.1914.

(±)-**CYY478**: R_f = 0.42 (ethyl acetate: *n*-hexane = 1:1); ^{1}H NMR (600 MHz, $CDCl_3$) δ 0.86 (d, *J* = 6.3 Hz, 3H, H-3'), 0.92 (d, *J* = 6.2 Hz, 3H, H-3'), 1.48-1.59 (m, 2H, H-2' and H-1'), 1.74-1.81 (m, 1H, H-1'), 2.49-2.58 (m, 3H, H-1, H-3, and H-6), 2.63 (dd, *J* =, 16.0, 3.1 Hz, 1H, H-7), 2.70 (dd, *J* = 11.6, 4.1 Hz, 1H, H-4_{ax}), 2.79 (ddd, *J* = 14.4, 3.3, 1.2 Hz, 1H, H-1), 2.96 (dd, *J* = 11.6. 2.0 Hz, 1H, H-4_{eq}), 2.98-3.01 (ddd, *J* = 11.3, 5.8, 1.2 Hz, 1H, H-6), 3.10-3.16 (m, 1H, H-7), 3.40 (d, *J* = 11.3 Hz, 1H, H-11b), 3.86 (s, 3H, 9-OMe), 5.54 (bs, 1H, 10-OH), 6.59 (s, 1H, H-8), 6.64 (s, 1H, H-11); ^{13}C NMR (150 MHz, $CDCl_3$) δ 22.5 (C-3'), 22.8 (C-3'), 25.9 (C-2'), 29.4 (C-7), 40.6 (C-1'), 45.1 (C-1) , 49.2 (C-3), 51.9 (C-6), 56.1 (-OMe), 59.7 (C-4), 62.0 (C-11b), 110.8 (C-11), 110.9 (C-8), 125.9 (C-7a), 129.8 (C-11a), 144.1 (C-9), 145.5 (C-10), 212.5 (C-2); HRESIMS calcd for $C_{18}H_{26}NO_3$ $[M+H]^+$, 304.1913; found, 304.1917.

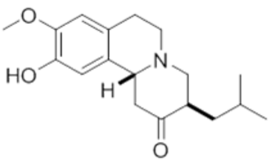

(+)-CYY477

Chemical Formula: $C_{18}H_{25}NO_3$
Exact Mass: 303.1834
Molecular Weight: 303.3960

(3*R*,11b*R*)-(+)-10-Hydroxy-3-isobutyl-9-methoxy-3,4,6,7-tetrahydro-1*H*-pyrido[2,1-*a*]isoquinolin-2(11b*H*)-one [(+)-CYY477]

To a solution of (±)-CYY477 (7.884 g, 25.99 mmol) in ethanol (165 mL), a solution of (+)-2,3-dibenzoyl tartaric acid monohydrate[5] (9.785 g, 26.00 mmol) in ethanol (150 mL) was added. The resulting mixture was stirred for 30 min at room temperature, then cooled to 6~10°C and stirred overnight. The suspension was centrifuged, supernatant removed, and evaporated to yield a white solid. The solid was recrystallized with ethanol (l g/ 20-30 mL) three times to give a white crystalline solid which was treated with CH_2Cl_2 and $NH_4OH_{(aq)}$ (0.1%, pH=10~11). The organic layers were washed with brine, dried over $MgSO_4$, filtered, and evaporated to afford (+)-CYY477 as a white solid (1.762 g, 5.81 mmol, 16%). $[\alpha]^{23}_{D}$ = +63.3^{o} (*c* = 0.335, MeOH); purity = 98.4% (RP-HPLC; Phenomenex Gemini C18); *ee* = 100% (chiral HPLC; AD-H); ^{1}H NMR (600 MHz, $CDCl_3$) δ 0.89 (d, *J* = 6.9 Hz, 3H, H-3’), 0.91 (d, *J* = 6.6 Hz, 3H, H-3’), 0.99-1.04 (m, 1H, H-1’), 1.62-1.68 (m, 1H, H-2’), 1.77-1.82 (m, 1H, H-1’), 2.32 (t, *J* = 11.7 Hz, 1H, H-4), 2.47-2.51 (m, 1H, H-1), 2.55-2.60 (m, 1H, H-6), 2.70-2.75 (m, 2H, H-7 and H-3), 2.85 (dd, *J* = 13.7, 3.1 Hz, 1H, H-1), 3.08-3.14 (m, 2H, H-6, H-7), 3.26 (dd, *J* = 11.6, 6.2 Hz, 1H, H-4), 3.45-3.47 (m, 1H, H-11b), 3.84 (s, 3H, 9-OMe), 5.73 (bs, 1H, 10-OH), 6.59 (s, 1H, H-8), 6.62 (s, 1H, H-11); ^{13}C NMR (150 MHz, $CDCl_3$) δ 22.2 (C-3’), 23.3 (C-3’), 25.5 (C-2’), 29.5 (C-7), 35.2 (C-1’), 47.4 (C-1) , 47.6 (C-3), 50.9 (C-6), 56.0 (-OMe), 61.5 (C-4), 62.4 (C-11b), 110.9 (C-8), 111.0 (C-11), 125.5 (C-11a), 129.5 (C-7a), 144.1 (C-10), 145.6 (C-9), 210.0 (C-2);

HRESIMS (*m*/*z*): $[M+Na]^+$ calcd. for $C_{18}H_{25}NNaO_3$, 326.1732; found, 326.1724; analysis (calc′d., found for $C_{18}H_{25}NO_3$): C (71.26, 71.08), H (8.31, 8.28), N (4.62, 4.32).

SUPPLEMENTARY DISCUSSION

We extended our behavioral findings in rodents to more complex behavioral actions of amphetamine, using a drug self-administration procedure in rats. The average response rates conformed to a bell-shaped function of dose for both methamphetamine and amphetamine self-administration, with comparable maximal response rates (0.086 ± 0.015 and 0.072 ± 0.012 responses/s, respectively; **Figs. 1c & d** and **Supplementary Fig. 2**). Rats self-administered cocaine at a maximum of 0.333 ± 0.143 responses/s (**Fig. 1e**). Thus, methamphetamine, amphetamine and cocaine treatments all reliably promoted self-administration. In contrast, neither (+)-CYY477 nor saline was self-administered at rates above those obtained when responses had no scheduled consequences (**Supplementary Figs. 2a & b**, compare open circles to points above EXT).

Acute pretreatment of rats with (+)-CYY477 decreased methamphetamine and amphetamine self-administration in a dose-dependent manner (**Figs. 1c & d**). Remarkably, at the highest (+)-CYY477 dose (0.01 mg/kg), amphetamine or methamphetamine self-administration was completely attenuated with response rates comparable to those obtained when responses had no scheduled consequences (**Figs. 1c & d**: points above EXT). Cocaine self-administration, in contrast, was unaffected by acute pretreatment with (+)-CYY477 (**Fig. 1e**). As a further control, we examined the effect of (+)-CYY477 on food reinforcement. (+)-CYY477 was equipotent in decreasing self-administration of methamphetamine and amphetamine and fully inhibited these responses at a dose that did not affect responses for food, suggesting that (+)-CYY477 specifically antagonized amphetamines without disrupting behavior maintained by other reinforcers (**Supplementary Fig. 2c**).

We also tested the ability of (+)-CYY477 to produce catalepsy and found that the (+)-CYY477 concentrations that blocked amphetamines' behavioral effects in our hyperlocomotion and drug self-administration assays were substantially lower than those required to induce catalepsy (**Supplementary Fig. 1b**) — likely through vesicular monoamine depletion[6, 7, 8]. We also note that different dosages of (+)-CYY477 were needed to block amphetamines' action in mouse locomotion and rat self-administration assays. This is most likely due to different psychostimulant dosing regimens and routes of administration (i.p. versus i.v.) as well as the different species used. Regardless, in both paradigms, acutely pre-administered (+)-CYY477 reproducibly blocked the behavioral actions of amphetamines but not of cocaine, which acts independently of VMAT2.

SUPPLEMENTARY METHODS

(+)-CYY477 Characterization

Details of the respective radioligand binding assays have been described previously[3, 9]. Brains were obtained from male Sprague-Dawley rats weighing 200-225 g, supplied on ice from Bioreclamation (Hicksville, NY) and subsequently dissected with the striatum removed and quickly frozen. Membranes were prepared by tissue homogenization in 20 volumes (w/v) of ice cold buffer using a Brinkman Polytron (setting 6, 20 s) and centrifuged at 30,000 x *g* (10 min, 4°C). For the DAT radioligand binding assay, the buffer was a modified sucrose phosphate buffer (0.32M sucrose, 7.74 mM Na_2HPO_4, 2.26 mM NaH_2PO_4, pH 7.4). For the VMAT2 radioligand binding assay, the buffer was a HEPES-sucrose buffer (50 mM HEPES, 0.32 M sucrose, pH 8.0). The resulting pellets were resuspended in buffer, centrifuged again and resuspended in buffer to a concentration of 10 mg/ml, original wet weight (o.w.w.). The DAT

radioligand binding assay was conducted in assay tubes containing 0.5 ml sucrose phosphate buffer for 120 min on ice. Each tube contained 0.5 nM [^{3}H]WIN 35,428 and 1 mg striatal tissue (o.w.w.). Nonspecific binding was determined using 0.1 mM (-)-cocaine HCl. The VMAT2 radioligand binding assay was conducted in polystyrene assay tubes containing 0.5 ml HEPES-sucrose buffer for 60 min at room temperature. Each tube contained 2 nM [^{3}H]dihydrotetrabenazine and 1 mg striatal tissue (o.w.w.). Nonspecific binding was determined using 20 μM (±)-tetrabenazine. Incubations for all binding assays were terminated by rapid filtration through Whatman GF/B filters presoaked in polyethylenimine (0.05% for [^{3}H]WIN 35,428 or 0.5% for [^{3}H]dihydrotetrabenazine), using a Brandel R48 filtering manifold (Brandel Instruments, Gaithersburg, MD). The filters were washed twice with 5 ml ice-cold buffer and transferred to scintillation vials. Cytoscint (MP Biomedical Solon, OH) (3.0 ml) was added and the vials were counted the next day using a Perkin Elmer Tri-Carb 2910 liquid scintillation counter (Perkin Elmer Health Sciences, Shelton, CT) at 50% efficiency. Similar binding assays were conducted using [^{3}H]citalopram or [^{3}H]nisoxetine to determine (+)-CYY477's binding affinities at the serotonin transporter (SERT) and the norepinephrine transporter (NET), respectively. K_i values were calculated using the Cheng-Prusoff equation[9].

FFN206 Characterization

FFN206 showed good specificity and low activity as desired for a false neurotransmitter. No submicromolar K_i values at any of the 62 CNS targets screened were observed. FFN206 was screened in radioligand binding assays for activity at 62 mammalian CNS receptors, channels and transporters by the Psychoactive Drug Screening Program (PDSP, UNC Chapel Hill, Dr. Bryan Roth) of the NIMH according to standardized protocols (see http://pdsp.med.unc.edu/).

FFN206 showed negligible affinity (K_i > 10 μM) at 59 targets and only weak affinity at three receptors: $5HT_{1B}$ (2.6 μM), adrenergic α_2 (2.6 μM), and muscarinic M_4 (4.7 μM). The targets surveyed were: $5HT_{1A,1B,1D}$, $5ht_{1e}$; $5HT_{2A,2B,2C}$; $5HT_3$, $5HT_4$, $5ht_{5a}$, $5HT_{6,7}$; Cholinergic nicotinic α2β2, α2β4, α3β2, α3β4, α4β2, α4β2**, α4β4 and Muscarinic $M_{1,2,3,4,5}$; Adrenergic $\alpha_{1A,1B,1D}$, $\alpha_{2A,2B,2C}$, $\beta_{1,2,3}$; Calcium channel (nitrendipine); Cannabinoid $CB_{1,2}$; Dopamine $D_{1,2,3,4,5}$; benzodiazepine (rat brain); $GABA_{A,B}$; δ, κ, μ,-opioid; Histamine $H_{1,2,3,4}$; $mGluR_5$ (rat brain); NMDA (PCP-binding site); Oxytocin; Peripheral benzodiazepine receptor; $Sigma_{1,2}$; $Vasopressin_{1A,1B,2}$; DAT; NET; SERT.

Rodent Husbandry

Animal care and maintenance were provided by a contract from the National Institute on Drug Abuse Intramural Research Program to Charles River Laboratories, Inc. (Wilmington, MA). The subjects were housed in humidity- and temperature-controlled colony rooms maintained on a 12:12-hour light:dark cycle with lights on at 07:00 hours. Subjects were acclimated to a temperature- and humidity-controlled vivarium for at least one week before use. Animals were cared for in accordance with all appropriate animal care guidelines according to the National Institutes of Health Animal Care and Use Program as well as the Animal Research: Reporting of In Vivo Experiments (ARRIVE) guidelines for reporting animal research. All procedures were approved by the institutional review board and ethics committee of the National Institute on Drug Abuse Intramural Research Program Animal Program, which is fully accredited by the Association for Assessment and Accreditation of Laboratory Animal Care (AAALAC) International. Moreover, all efforts were made to ameliorate animal suffering.

For experiments involving mice as subjects, we used male Swiss-Webster mice (Charles River Laboratories, Inc.) weighing 30 to 35 g which were approximately 8 weeks of age. Mice were housed in groups of 3-4 per cage with food and water available at all times. For experiments involving rat subjects, we used 18 male Sprague-Dawley rats (Taconic Biosciences, Germantown, NY) weighing ~300 g and approximately 8 weeks of age on receipt. Rats were singly housed with water and initially food available at all times. All subjects were acclimated to the animal colony for a minimum of one week before use. After acclimation, body weights of rats were maintained at ~320 g by adjusting daily food rations (Scored Bacon Lover Treats, Bio-Serv, Frenchtown, NJ).

Mouse Catalepsy

Each mouse was removed from its cage and injected with either (+)-CYY477, tetrabenazine, haloperidol, or vehicle and replaced in its cage. 30 min after injection, each mouse was again removed from its respective cage, and gently placed on the laboratory bench with its forepaws on a 0.5 cm diameter bar measuring 11 cm in length and positioned 4 cm above the bench. Catalepsy was considered present when the forepaws remained on the bar, as that position is not a natural posture for a mouse to assume. The intensity of the cataleptic state was assessed by measuring the duration of that posture, which ended when the mouse first moved its forepaws off of the bar or moved its head in an exploratory manner. If the subject remained immobile for 100 s, it was removed and placed back in its cage. Observations were also made at 60, 90 and 120 min after injection. Treatments were administered to 4 mice no more frequently than once every 3 days, and each mouse received all treatments.

Mouse Locomotion Assay

Male Swiss-Webster mice (N=132) were placed singly in clear acrylic chambers (43.2 x 43.2 x 29.8 cm) for the assessment of horizontal locomotor activity. The acrylic chambers fit within monitors (Med Associates, St. Albans, VT), which were equipped with detectors sensitive to infrared light which were spaced 2.5 cm apart along two perpendicular walls. Mounted on the opposing walls and directed at the detectors were infrared light sources. One activity count was registered each time the subject interrupted a single light beam. White noise was present in the room throughout testing to mask extraneous sounds. Ambient illumination was provided by overhead illumination of the laboratory room. Mice were injected with (+)-CYY477 (i.p. in volumes of 1.0 ml/100 g) and then immediately injected with psychostimulants and placed in the apparatus, with activity counts totaled each 10 min over a 30 min period. Each treatment was administered to 6 mice and every mouse was used once.

Rat Drug Self-Administration

Male Sprague-Dawley rats (N=18) served as subjects. Experimental sessions were conducted daily with subjects placed in operant-conditioning chambers (modified ENV-203, Med Associates, St. Albans, VT) that measured 25.5 x 32.1 x 25.0 cm and were enclosed within sound-attenuating cubicles equipped with a fan for ventilation and white noise to mask extraneous sounds. On the front wall of each chamber were two response levers, 5.0 cm from the midline and 4.0 cm above the grid floor. A downward displacement of a lever with a force approximating 0.20 N defined a response and always activated a relay mounted behind the front wall of the chamber producing an audible “feedback” click. 3 light-emitting diodes (LEDs) were located in a row above each lever. A receptacle for the delivery of 20 mg food pellets (Bio-Serv,

Rodent Purified Diet, Grain-Based Dustless Precision Pellets) via a pellet dispenser (Med Associates, Model ENV-203-20), was mounted on the midline of the front wall between the two levers and 2.0 cm above the floor. A syringe infusion pump (Model 22, Harvard Apparatus, Holliston, MA) placed above each sound-attenuating cubicle delivered injections of specified volumes from a 10 ml syringe. The syringe was connected by Tygon tubing to a single-channel fluid swivel (375 Series Single Channel Swivels, Plymouth Meeting, PA) mounted on a balance arm above the chamber. Tygon tubing from the swivel to the subject's catheter was protected by a surrounding metal spring and completed the connection to the subject.

Drug naïve rats (N=12) were surgically implanted under anesthesia (ketamine 60.0 mg/kg, i.p. and xylazine 12.0 mg/kg, i.p.) in the right external jugular vein with a chronic indwelling catheter that exited at the mid-scapular region of the animal's back. Catheters were infused daily with 0.1 ml of a sterile saline solution containing heparin (30.0 IU/ml) and penicillin G potassium (250,000 IU/ml) to minimize the likelihood of infection or clot and fibroid formation. All animals were allowed to recover from surgery for approximately 7 days before drug self-administration studies were initiated.

Experimental sessions initially lasted for 120 min. Sessions started with illumination of the LEDs above each lever. Each downward deflection of the right lever turned off the LEDs, produced an audible click, and activated the infusion pump for a 10 s (fixed-ratio or FR one schedule) drug injection of either methamphetamine (0.1 mg/kg/injection, N=6), or cocaine (0.32 mg/kg/injection, N=6) followed by a 20 s time-out period during which LEDs were off and responding had no scheduled consequences. After the time-out, the LEDs were illuminated with

subsequent responses having the scheduled consequences. Responses on the left lever were recorded but had no scheduled consequences. Subjects were returned to their home cages in the vivarium after each session. The procedure was then modified to facilitate assessments of a range of self-administered drug doses. Consequently, the ratio of responses to injections (with accompanied stimulus changes) was changed to a FR 5 schedule with sessions subsequently divided into five 20 min components, each preceded by a 2 min time-out period. During the time-out period, all lights within the chamber were off and responses had no scheduled consequences. By adjusting infusion volumes and durations, the drug dose per injection was incremented in each sequential component as follows: no injection (also referred to as extinction, or EXT, because responses had no scheduled consequences) followed by 0.01, 0.03, 0.10, and 0.32 mg/kg/injection for methamphetamine, and EXT, 0.03, 0.10, 0.32, or 1.0 mg/kg/injection for cocaine based on a body weight of 0.32 kg. Infusion volumes (and durations) for the sequential drug administration components were as follows: 0 μl/0 s, 5.6 μl/0.32 s, 18.0 μl/1.0 s, 56.0 μl/3.2 s, and 180 μl/10.0 s. A sample injection of the respective drug at the corresponding dose occurred independently of subject response just before the beginning of each component (except the first component during which there were no injections). These conditions remained until response rates demonstrated less than 20% variation across three consecutive sessions. Once performance responses were stable, various compounds were substituted for each of the original self-administered drugs to assess drug effects on i.v. self-administration. The compounds substituted in half-log dose increments for either methamphetamine or cocaine were: amphetamine (0.01, 0.032, 0.1 and 0.32 mg/kg/injection), (+)-CYY477 (low-dose range: 0.032, 0.1, 0.32 and 1.0 μg/kg/injection; high-dose range: 1.0, 3.2, 10.0, and 32.0 μg/kg/injection), and saline for subjects trained with methamphetamine. Effects of pre-session (+)-CYY477 injections

(i.p.) on i.v. self-administration of methamphetamine, amphetamine, or cocaine were assessed by injecting (+)-CYY477 5-min before selected sessions. At least 72 hours separated tests of (+)-CYY477, which were conducted in a mixed order of doses. Response rates were determined by dividing responses by appropriate elapsed times (excluding time outs and 0.32-10 s for each drug injection).

To assess (+)-CYY477's selectivity of effect for drug self-administration, responses were maintained by different amounts of food in a separate cohort of subjects (N=6) using a procedure similar to the drug self-administration schedule described above. All conditions were identical to those used with drug self-administration except that food pellet presentations replaced the injections. Briefly, experimentally naïve subjects were trained with food reinforcement (20 mg grain-based food pellets, Bio-Serv) to press the right lever under an FR 5-response schedule of reinforcement. After training, the procedure was modified to a 5-component procedure analogous to that used for drug self-administration with different numbers of food pellets (0-4 pellets) delivered for completion of each FR 5 in successive components. Subjects were fed their daily food ration (~35 g of 1 g chocolate-flavored pellet, Bio-Serv) 150 min before sessions so that their response rates approximated those maintained by drug injections. Once performances were stable (as defined above), the effects of pre-session (+)-CYY477 injections (i.p.) were assessed by injecting (+)-CYY477 5-min before selected sessions. At least 72 hours separated tests of (+)-CYY477, which were conducted in a mixed order of doses. Response rates were determined by dividing responses by appropriate elapsed times (excluding time outs and 100 ms for each food presentation).

SUPPLEMENTARY REFERENCES

1. Kenney C, Jankovic J. Tetrabenazine in the treatment of hyperkinetic movement disorders. *Expert Rev. Neurother.* **6**, 7-17 (2006).
2. Kilbourn MR, Lee LC, Heeg MJ, Jewett DM. Absolute configuration of (+)-α-dihydrotetrabenazine, an active metabolite of tetrabenazine. *Chirality* **9**, 59-62 (1997).
3. Yao Z. *et al*. Preparation and evaluation of tetrabenazine enantiomers and all eight stereoisomers of dihydrotetrabenazine as VMAT2 inhibitors. *Eur J Med Chem* **46**, 1841-8 (2011).
4. Magyar Tudomanyos Akademia Process for the production of quinolizine derivatives. *GB 958936 (A)*, May 27, **1964**.
5. Duffield AJ, Johnston G, Green BM, Townsley JC. Desmethyl derivatives of tetrabenazine and pharmaceutical compositions thereof. *WO2010026436 A2*, March 11, 2010.
6. Korner G *et al.* Brain catecholamine depletion and motor impairment in a Th knock-in mouse with type B tyrosine hydroxylase deficiency. *Brain* **138**, 2948-2963 (2015).
7. Fuenmayor LD, Vogt M. The influence of cerebral 5-hydroxytryptamine on catalepsy induced by brain-amine depleting neuroleptics or by cholinomimetics. *British journal of pharmacology* **67**, 309-318 (1979).
8. Fuenmayor LD, Vogt M. Production of catalepsy and depletion of brain monoamines by a butyrophenone derivative. *British journal of pharmacology* **67**, 115-122 (1979).
9. Hiranita T, *et al.* 2-Isoxazol-3-Phenyltropane Derivatives of Cocaine: Molecular and Atypical System Effects at the Dopamine Transporter. *J Pharmacol Exp Ther,* (2014).
10. Cheng Y, Prusoff WH. Relationship between the inhibition constant (K1) and the concentration of inhibitor which causes 50 per cent inhibition (I50) of an enzymatic reaction. *Biochemical pharmacology* **22**, 3099-3108 (1973).
